# A two-stage Bayesian method for estimating accuracy and disease prevalence for two dependent dichotomous screening tests when the status of individuals who are negative on both tests is unverified

**DOI:** 10.1186/1471-2288-14-110

**Published:** 2014-09-23

**Authors:** Jin Liu, Feng Chen, Hao Yu, Ping Zeng, Liya Liu

**Affiliations:** Department of Epidemiology and Biostatistics, School of Public Health, Nanjing Medical University, Nanjing, Jiangsu PR China

**Keywords:** Bayesian modeling, Screening test, Test accuracy, Disease prevalence, Model checking

## Abstract

**Background:**

Estimating the disease prevalence and test accuracy (sensitivity and specificity) for two dependent screening tests when the status of individuals who are negative on both tests is unverified represents a considerable challenge, as the disease rates for individuals negative on both tests are not identifiable without additional assumptions.

**Methods:**

This article presents a unified framework for handling this non-identifiability problem using two-step hierarchical informative prior on the sensitivities by two-stage Bayesian modeling with the characterized by joint testing strategies based on the inherent attribute of screening/diagnostic tests. We assign a diffuse and less risky two-step hierarchical informative uniform prior to the sensitivities while assigning a uniform (0,1) prior distribution to the specificities and prevalence. Strategies for model evaluation, general global evaluations, and individual cell checking are presented. Simulations are conducted under various scenarios to evaluate the performance of the proposed method. Applications to real data are also presented to illustrate the potential impact and benefit of the proposed method.

**Results:**

Our results indicate that when the priors of sensitivities are assigned as appropriate two-step hierarchical informative priors, or even in the absence of the priors for the specificities and prevalence, the parameters involved in this study can still be estimated well. The advantages and limitations of this method in solving such problems are discussed and compared with other two-stage methods.

**Conclusions:**

We developed a two-stage Bayesian method for two dependent dichotomous screening tests with unverified individuals who are negative on both tests, and addressed the ad hoc model evaluation and checking procedures. The method can be understood easily and used conveniently by non-statisticians.

**Electronic supplementary material:**

The online version of this article (doi:10.1186/1471-2288-14-110) contains supplementary material, which is available to authorized users.

## Background

Screening programs for a specific disease or condition of interest are typically divided into two stages. In the first stage, a population of known size *n* is screened by simple and rapid but imperfect tests to classify people as likely or unlikely to have the disease or condition of interest. In the second stage, individuals who appear likely to have the disease or condition are examined further using a gold standard test to confirm the disease or condition. To improve the sensitivity of these screenings, two simple and rapid tests are often used in the first stage [1]. If either of the tests is positive, then a full evaluation of the correct disease status using a gold standard classification is undertaken in the second stage [2]. This strategy is widely used in screening for chronic diseases, infectious diseases, and animal diseases [2–4]. One characteristic of this strategy is that negatives in both tests are not verified by a gold standard test because the disease probability in the sub-population is so low that further investigations are costly, unacceptable, and possibly unethical. Another characteristic is that the two tests in the first stage are often conditionally dependent on disease status and/or non-disease status [5]. If the two screening tests have a similar biological basis, as is often the case, the conditional dependence assumption is suitable. For example, when we use two fecal occult blood tests (occult blood tests and immunohistochemical methods) for colon cancer screening, a positive occult blood test is often accompanied by a positive immunochemical result; thus, the results of the two tests are dependent [2].

Estimating the disease prevalence and test accuracy (sensitivity and specificity) when the two tests are dependent and when individuals negative on both tests are unverified is a considerable challenge, as the disease rate for individuals who are negative on both tests is not identifiable without additional assumptions [5–9]. Li et al. reviewed several methods for resolving the non-identifiability problem in the framework of classical statistics [8]. They classified these methods into two types. The methods of the first type are intended to solve the non-identifiability problem by introducing additional assumptions about the association of the two tests. Walter et al. assumed that the two test errors were independent [9], while van der Merwe also assumed that the two tests were independent under the disease status but dependent under non-disease status [10]. The methods in the second case resolve the non-identifiability problem directly by adding an assumption that is more likely to hold than the independence assumption [7]. Bohning and Patilea proposed a capture-recapture approach for screening using two diagnostic tests with information on disease status available for the test positives only [11]. Chu et al. proposed latent class models for screening studies using two screening tests, with a categorical disease status verified in positives test only [7]. However, these studies did not provide sufficient insight and details to solve the problem [8]. Li et al. presented a unified framework for solving the non-identifiability problem by saturating the model using certain homogeneous association assumptions [8]. In their paper, although the five distinct models provided different estimators, they were all saturated models and provided the same maximum likelihood. Therefore, they are not differentiable in terms of goodness-of-fit [8].

Recently, Bayesian analysis has been used increasingly to solve the non-identifiability problem using prior information [12–17]. Briefly, the use of this analysis is appropriate for three scenarios. The first involves the use of Bayesian model averaging (BMA) to address this challenging estimation problem, as a different homogeneous-dependent model can provide the same goodness-of-fit for the data but with substantively different estimates [7]. The second scenario involves the application of BMA to inferences over conditional independence and dependence models for the estimation of disease prevalence in a situation involving two imperfect tests in the absence of a gold standard [18]. The third scenario involves the administration of a moderately effective but relatively cheap screening test to all subjects in the first stage, after which a gold standard test is performed on a subset of the high screen and low screen groups in the second stage [19].

This article presents a unified framework to handle the non-identifiability problem by a two-stage Bayesian model using two-step hierarchical informative uniform prior on the sensitivities. We provide detailed Bayesian modeling for stages one and two, the specification of the prior distribution, and the calculation of the posteriors of the parameter distributions. The strategies for model evaluation and checking are proposed. We illustrate our approach using an example and simulation and conclude this article with a discussion and some remarks.

## Methods

### Two-stage Bayesian modeling

#### Data structure and estimated parameters

Table 1 presents the outcomes of two screening tests (*T*_*1*_*, T*_*2*_) from a total of *n* subjects, with disease statuses (*D*_*+*_*, D*_*-*_) verified only for individuals with at least one positive test. *D*_*+*_ represents a true diseased condition, whereas *D*_*-*_ represents the non-diseased, as determined by a gold standard test. *T*_*i*(+,-)_ (*i* = 1,2) represents the results of the test *i*. Let vector *x* = (*x*_*11*_, *x*_*10*_, *x*_*01*_, *x*_*00*_) denote the observed number for each combination of two test outcomes (1 or 0 for test positive or test negative). Let *SI*_(+,-)_ (abbreviation of *Si*multaneous testing) represent the outcomes of the simultaneous testing in stage one, and let *a*_*11,*_*a*_*10,*_*a*_*01,*_*a*_*00*_ denote *D*_*+*_*SI*_*+*_*, D*_*-*_*SI*_*+*_, *D*_*+*_*SI-*, *D- SI-*, respectively. Because the full evaluation of the disease status is not performed if neither of the two tests is positive in the first stage, the frequencies of [*a*_*01*_] and [*a*_*00*_] in the bracket are unobserved, whereas the total frequency of both tests being negative *a*_*0****.***_ *=* [*a*_*01*_] + [*a*_*00*_] is known.

**Table 1:** **Data structure of two-stage Bayesian modeling for two dependent dichotomous screening tests with individuals who are negative in both tests unverified**

	Stage one				Stage two		
	T _2+_	T _2-_	Total		D+	D-	Total
T_1+_	x_11_	x_10_	x_1._	SI_+_	a_11_	a_10_	a_1._
T_1-_	x_01_	x_00_	x_0._	SI_-_	[a_01_]	[a_00_]	a_0._
Total	x_.1_	x_**.**0_	n	Total	[a_.1_]	[a_**.**0_]	n

*Note*: SI, simultaneous testing; [] indicates that the frequencies are unavailable.

The prevalence is defined as *π* = *P*(*D+*). The sensitivity and specificity for the *i*th test are defined as *Se*_*i*_ *= P(T*_*i+*_*|D*_*+*_*)* and *Sp*_*i*_ *= P(T*_*i-*_*|D*_*-*_*)*, respectively. Furthermore, let *CovDp* and *CovDn* be the covariances between the two tests for diseases (*CovDp*) and for non-diseases (*CovDn*). Let *ρ*_*D+*_ and *ρ*_*D-*_ be the correlation coefficients under the conditions of disease and non-disease, and let *PPV*_*JE*_ be the positive predictive value of the simultaneous testing. These parameters are defined as follows [12, 20, 21]:
1

where *Se*_*JE*_ and *Sp*_*JE*_ denote the joint sensitivity and specificity of the simultaneous testing (described in equation (6))
2

where *Se*_11_ = *P*(*T*_1 +_, *T*_2 +_|*D*_+_), *Sp*_22_ = *P*(*T*_1 -_, *T*_2 -_|*D*_-_)
3

#### Bayesian modeling for stage one

According to the screening programs, in the first stage (see Table 1, left column), two simple and rapid but potentially imperfect screening tests, denoted as *T*_*1*_ and *T*_*2*_, are applied simultaneously to each of the units sampled. If either individual test is positive, this condition indicates a positive result of the joint test. The strategy is often called simultaneous testing and seeks to obtain a joint testing strategy with increased sensitivity [22]. Let vector *Px* = (*Px*_*11*_*,Px*_*10*_*,Px*_*01*_*,Px*_*00*_) be the probabilities, respectively, from the vector *x* = (*x*_*11*_, *x*_*10*_, *x*_*01*_, *x*_*00*_). Supposing that *n* subjects are sampled randomly with prevalence *π*, the vector *x* has the following multinomial sampling distribution:
4

Considering that the two tests are conditionally dependent under disease status and non-disease status, the multinomial cell probabilities are given by
5

In stage one, the individuals are classified as likely to have the disease or condition of interest only if either of the two test outcomes is positive. The joint sensitivity and specificity of the simultaneous testing (denoted by the subscript JE, for ‘Joint Either’) are as follows:
6

#### Bayesian modeling for stage two

In the second stage, a gold standard test is used only for positive samples from the simultaneous testing of the first stage, whereas the negative samples are not verified by the gold standard test (see the right-hand column in Table 1). Obviously, in this case, the simultaneous testing of the first stage and the gold standard testing of the second stage constitute sequential testing [22]. This sequential testing is used mainly to increase feasibility because applying the gold standard test to the negatives is often costly, unacceptable, and unethical. Given that the gold standard test and the simultaneous testing are independent and that the sensitivity and specificity of the gold standard test are equal to 100%, the joint sensitivity and specificity of the sequential testing (denoted by the subscript JB, for ‘Joint Both’) are as follows:
7

where *Se*_*GS*_ and *Sp*_*GS*_ are the sensitivity and specificity of the gold standard (denoted by the subscript GS, for ‘*G*old *S*tandard’), respectively.

Let vector *a* = (*a*_*11*_*, a*_*10*_*, a*_*0.*_) denote the observed number for each combination in stage two, where *a*_*0.*_ is the sum of the test negatives from the first stage. Let *Pa* = (*Pa*_*11*_*,Pa*_*10*_*,Pa*_*0.*_) represent the respective probabilities from vector *a*. Consider that elements from the proportion vector of multinomial *a* are required the probabilities to sum to 1. The multinomial sampling distribution is given by
8

where the multinomial cell probabilities are given by
9

#### Prior distributions

For Bayesian statistics, we wish to use all available information at the design stage but might prefer a more vague, less risky prior at the data analysis stage [23]. To reduce the influence of subjective opinions on the data analysis as much as possible, we placed an informative prior distribution on a minimum number of parameters [24].

In principle, a uniform prior distribution or Beta prior distribution can be used over the set of unknown parameters. We chose the uniform non-Beta prior distribution because a uniform prior distribution is more easily accessible for non-statisticians and less risky in this study. For example, to determine the value of *α* and *β* for a Beta prior distribution, we need to know the corresponding mean and standard deviation or the 2.5 and 97.5 percentiles [25], whereas for a uniform prior distribution, we need only to determine the range of estimated parameters. Especially for the main parameters (*Se*_*1*_*, Se*_*2*_*, Sp*_*1*_*, Sp*_*2*_*,*) in this study, lower bound values for the uniform distribution are often estimated securely based on expert opinion, published papers, or even test kit instructions. For example, an epidemiologist can easily be assured that the sensitivity of the enzyme-linked immunosorbent assay (ELISA) for HIV antibody screening is higher than 80% [4]; i.e., the lower bound of the uniform distribution is 0.8. However, a reasonable choice for the upper bound of a uniform prior distribution is sometimes difficult to determine because the accuracy of the test kit might differ under various practical conditions. We use the method of two-step hierarchical priors to set the upper bound of the uniform distribution. For an estimated parameter, such as sensitivity (*Se*_*i*_), we first provide a uniform prior distribution (*a*_*Sei*_*, b*_*Sei*_) and then give *b*_*Sei*_ another uniform distribution (*b*_*1Sei*_*, b*_*2Sei*_). The method of two-step hierarchical priors is a good strategy if it is difficult to determine the prior distribution using a one-step method; even though an incorrect prior is set in step two, the risk of this action resulting in a mistaken result is smaller than in a one-step prior [26].

For sensitivity, we set two steps for the hierarchical uniform priors:
10

For specificities and prevalence, we might set a uniform prior distribution based on the characteristics of this type of study (see the Discussion section):
11

For covariances *CovDp and CovDn,* the feasible range is determined by the sensitivities among the diseased subjects and the specificities among the non-diseased subjects, where *0* ≤ *CovDp* ≤ min (*Se*_*1*_*,Se*_*2*_)*Se*_*1*_*Se*_*2*_ for the diseased subjects and *0* ≤ *CovDn* ≤ min(*Sp*_*1*_*,Sp*_*2*_)*Sp*_*1*_*Sp*_*2*_ for the non-diseased subjects [20]. Because prior information regarding the two covariances is typically unavailable, uniform prior distributions over these ranges can be used for *CovDp* and *CovDn*:
12

#### Calculation of the posteriors of parameter distributions

The posteriors of parameter distributions were calculated using Markov-chain Monte Carlo techniques, in particular the Gibbs sampler in WinBUGS (MRC Biostatistics Unit, Cambridge, UK) [27]. For the analyses presented in this paper, inferences were based on 105,000 iterations after discarding an initial burn-in of 5,000 iterations, with convergence assessed by running multiple chains from various starting values [28]. The WinBUGS code used in this paper is available in the Additional files 1 and 2 and can be altered easily for use with different data.

### Model evaluation and checking

Model evaluation and checking are highly active areas of Bayesian statistics research. Researchers can use various statistics to determine the plausibility of an assumption of interest in light of the observed data [29]. In this study, we divide the model evaluation and the checking of the assumptions into individual and overall diagnostics. Individual checks are based on the cell in Table 1, and overall diagnostics aim to check the more general assumptions of the model by *DIC* [21], *pD* [5], and the Chi-squared goodness-of-fit test. The technical details were given in Additional file 3. Given the special circumstance that the negatives for both tests are unavailable, none of the methods could be used alone in the preceding model checks because they diagnosed the models from a different perspective. For example, a local Chi-squared goodness-of-fit test is based only on known cells. For this reason, it is recommended that the above methods be combined for model checking.

Below, we present an ad hoc preliminary, exploratory model that serves to check the criteria, based on empirical information for this type of study. If (1) the values of *DIC* and *pD* are reasonable [5]; (2) the *p*-value of the local *χ*^*2*^ test is close to 0.5 or far from zero or one [29]; (3) the 95% *BCI* of the estimated *PPV* (model key point) includes its actual value; and (4) most frequencies of the cells in Table 1 fall within their corresponding Bayesian credible intervals, we may conclude that the model fits the data well based on the current information and at a specified probability level.

## Results and discussion

### Results

#### Screening study for colorectal cancers

The data in Table 2 are based on the data of Castiglione et al. [30]. That study compared rehydrated guaiac testing (Hemoccult) on three consecutive bowel movements with immunochemical testing using reversed passive haemagglutination (RPHA—Hemselect) on the first bowel movement only to detect colorectal cancers or adenomas ≥ 10 mm in 5727 individuals aged 40–59 years. Subjects with a positive hemoccult and/or a positive/borderline hemeselect test were invited to undergo pancolonoscopy. A double-contrast barium enema was performed when pancolonoscopy was not possible. Further details of the methods used in the study were provided by Castiglione et al. [2], and the data were analyzed by Geoffrey Berry et al [6].

**Table 2:** **Observed frequencies in the screening study for colorectal cancers in 5727 individuals aged 40–59 years** [6]

	Stage one				Stage two		
	T _1+_	T _1-_	Total		D+	D-	Total
T_2+_	39	91	130	SI_+_	29	338	367
T_2-_	237	5360	5597	SI_-_	[a_01_]	[a_00_]	5360
Total	276	5451	5727	Total	[a_**.**1_]	[a_**.**0_]	5727

*Note*: T_1_, rehydrated Hemoccult; T_2_, RPHA-Hemeselect; SI, simultaneous testing; [] indicates that the frequencies are unavailable.

According to the description in of *Prior distribution* section of this paper, we placed hierarchical uniform priors on *Se*_*1*_ and on *Se*_*2*_. These hierarchical priors were elicited based on the opinions of a co-author (Dr. Hao Yu) and on published papers [2, 30]. The expert opinion and previous information showed that the sensitivity of test 1 (rehydrated hemoccult) was less than the sensitivity of test 2 (RPHA-Hemeselect) and that the values of their lower bounds values were at least 0.5 and 0.6, respectively. The values of the upper bounds for *Se*_*1*_ and *Se*_*2*_ were assigned as much diffuse, less risky two-step hierarchical priors, as follows:


For the specificities and prevalence, we set the following uniform prior:


For the covariances *CovDp* and *CovDn*, the uniform priors were assigned according to equation (12).

It is clear from Table 3 that the results from the two-stage Bayesian model with two-step hierarchical prior on *Se*_*1*_ and *Se*_*2*_ fit the data well in accordance with the criteria presented in the *Model evaluation and checking* section. All of the estimated values were close to their true values, and all fell within 95% Bayesian credible intervals over their corresponding true values. The effective number of parameters, a *pD* of approximately 3.9, indicates that the two-stage Bayesian strategy substantially improves the identifiability of the models. The *χ*^*2*^ *=* 0.002 and *P* = 0.61 values indicate that the distributions of the replicated and actual data are similar.

**Table 3:** **Posterior parameters and models checking for the application of two-stage Bayesian models to the colorectal cancers data**

Posterior parameters		Model evaluation and checking		
Median (95% BCI)		True value Median (95% BCI)		
π	0.0065 (0.0043, 0.0095)	PPV_JE_	0.08	0.0807 (0.0559, 0.1107)
Se_1_	0.6040 (0.5048, 0.8834)	x_11_	39	39 (24, 58)
Se_2_	0.6825 (0.6039, 0.9036)	x_10_	91	91 (67, 117)
Sp_1_	0.9812 (0.9772, 0.9849)	x_01_	237	237 (200, 277)
Sp_2_	0.9559 (0.9513, 0.9603)	x_00_	5360	5359 (5314, 5402)
Se_JE_	0.8103 (0.6503, 0.9557)	a_11_	29	30 (16, 46)
Sp_JE_	0.9405 (0.9360, 0.9450)	a_10_	338	338 (296, 382)
			pD = 3.9	DIC = 39.7
			*χ* ^2^ = 0.0002	P = 0.6101

Table 3 shows the sensitivity and specificity of joint testing to be approximately 0.81 and 0.94, respectively, with the strategy of joint-simultaneous testing adding approximately 0.13 (*Se*_*JE*_ (0.8103) minus *Max*(*Se*_*1*_, *Se*_*2*_) (0.6825)) to the sensitivity at the expense of only an approximate loss of 0.04 (*Sp*_*JE*_ (0.9405) minus *Max*(*Sp*_*1*_, *Sp*_*2*_) (0.9812)) in specificity for the colorectal cancer data. This result conformed to the theory of joint-simultaneous testing. The correlation coefficients under disease and non-disease status, which were approximately 0.4 and 0.1, respectively, suggested moderate and weak dependence under the conditions of disease and non-disease, respectively.

To analyze the influence of the prior distribution of sensitivities (*Se*_*i*_, (*i* = 1,2)) on posterior distributions, the non-informative prior, pessimistic and optimistic two-step hierarchical informative priors were assigned, respectively, as below:


For the specificities and prevalence, the following uniform priors were assigned:


Table 4 shows that prior information of the sensitivities produced substantive influence on the posterior distributions. The hierarchical informative priors on the sensitivities must be assigned reasonably in these types of studies.

**Table 4:** **Influence of the prior distributions of sensitivities on posterior distributions**

	Se _i_ ~ U(0, 1)	Se _i_ ~ U(0.1, U(0.2, 0.99))	Se _i_ ~ U(0.8, U(0.9, 0.99))
	Median (95% BCI)	Median (95% BCI)	Median (95% BCI)
π	0.0102 (0.0044, 0.1793)	0.0130 (0.0056, 0.0323)	0.0055 (0.0038, 0.0075)
Se_1_	0.3053 (0.0075, 0.9666)	0.2461 (0.1065, 0.1065)	0.8691 (0.803, 0.9612)
Se_2_	0.2658 (0.0055, 0.9397)	0.2414 (0.1057, 0.7268)	0.8675 (0.8032, 0.9596)
Sp_1_	0.9805 (0.9740, 0.9851)	0.9806 (0.9762, 0.9848)	0.9818 (0.9781, 0.9853)
Sp_2_	0.9540 (0.9434, 0.9595)	0.9545 (0.9494, 0.9594)	0.9561 (0.9516, 0.9604)
Se_JE_	0.5162 (0.0293, 0.9861)	0.3996 (0.1703, 0.8295)	0.9466 (0.8561, 0.9899)
Sp_JE_	0.9398 (0.9276, 0.9445)	0.9401 (0.9354, 0.9446)	0.9405 (0.9360, 0.9449)

Note: U, uniform distribution; BCI, Bayesian credible interval.

Berry et al. analyzed the data using maximum likelihood by fitting the four versions of the models: (1) the independence model, (2) independence in the non-diseased group, (3) independence in the diseased group, and (4) the dependence model [6]. Based on the *χ*^*2*^ test statistics, our models provide a better fit to the data than those in the Berry et al. paper. In addition, our method can estimate parameter intervals that were not reported in the Berry et al. paper. In fact, it is difficult for the method of Berry et al. to calculate the confidence intervals of certain complex statistics involved in joint simultaneous testing, such as the joint positive predictive value (*PPV*_*JE*_), joint sensitivity (*Se*_*JE*_), and joint specificity (*Sp*_*JE*_), which are important in evaluating model fitting in this type of study.

#### Simulation studies

To further illustrate the performance of the two-stage Bayesian models, we applied them to a series of simulated data sets with a conditional correlation coefficient of *ρ*_*D+*_ = 0.5 for the diseased and *ρ*_*D-*_ = 0.4 for the nondiseased, as would typically be found in practice. Because our models contain a large number of parameters and because the study design has been associated with a wide range of prevalence values, sample sizes, and test properties, it is impossible to investigate the performance of these models across all possible scenarios. We therefore selected a range related to the actual screening and diagnostic tests, based on the following parameters:

(1) low prevalence and large sample size, such as cancer screening for community population [30] and HIV-antibody screening for blood donors [31].

(1.1) high test accuracy, (*π*, *n*, (*Se*_1_, *Sp*_1_), (*Se*_2_, *Sp*_2_)) = (0.01, 20000, (0.90, 0.95), (0.95, 0.90));

(1.2) low test accuracy, (*π*, *n*, (*Se*_1_, *Sp*_1_), (*Se*_2_, *Sp*_2_)) = (0.01, 20000, (0.60, 0.70), (0.70, 0.60)).

(2) high prevalence and small sample size, such as the screening of suspicious patients in the hospital or clinic.

(2.1) high test accuracy, (*π*, *n*, (*Se*_1_, *Sp*_1_), (*Se*_2_, *Sp*_2_)) = (0.40, 200, (0.90, 0.95), (0.95, 0.90));

(2.2) low test accuracy, (*π*, *n*, (*Se*_1_, *Sp*_1_), (*Se*_2_, *Sp*_2_)) = (0.40, 200, (0.60, 0.70), (0.70, 0.60))

We also examined the consequences of specifying different prior information, which were divided into the four scenarios (Table 5) given below:

**Table 5:** **Priors of two-stage Bayesian modeling for simulation studies**

True value	Informative priors for model	Informative priors for Se alone	Two-step informative priors for Se alone	Informative priors for Sp alone
Se = 0.6	U(0.5,0.7)	U(0.5,0.7)	U(0.5,U(0.55,0.99))	U(0,1)
Se = 0.7	U(0.6,0.8)	U(0.6,0.8)	U(0.6,U(0.65,0.99))	U(0,1)
Se = 0.8	U(0.7,0.9)	U(0.7,0.9)	U(0.7,U(0.75,0.99))	U(0,1)
Se = 0.9	U(0.85,0.95)	U(0.85,0.95)	U(0.85,U(0.9,0.99))	U(0,1)
Se = 0.95	U(0.90,0.99)	U(0.90,0.99)	U(0.9,U(0.95,0.99))	U(0,1)
Sp = 0.6	U(0.5,0.7)	U(0,1)	U(0,1)	U(0.5,0.7)
Sp = 0.7	U(0.6,0.8)	U(0,1)	U(0,1)	U(0.6,0.8)
Sp = 0.8	U(0.7,0.9)	U(0,1)	U(0,1)	U(0.7,0.9)
Sp = 0.9	U(0.85,0.95)	U(0,1)	U(0,1)	U(0.85,0.95)
Sp = 0.95	U(0.90,0.99)	U(0,1)	U(0,1)	U(0.90,0.99)
π = 0.4	U(0.3,0.5)	U(0,1)	U(0,1)	U(0,1)
π = 0.1	U(0.05,0.15)	U(0,1)	U(0,1)	U(0,1)
π = 0.01	U(0.005,0.015)	U(0,1)	U(0,1)	U(0,1)

*Note*: π, prevalence; U, uniform distribution.

1. informative priors for model: the prior density of the estimated model parameters (*Se*_*1*_*, Se*_*2*_*, Sp*_*1*_*, Sp*_*2*_*,* and *π*) was centered at their true values.
2. informative priors for sensitivities alone: the prior density of the sensitivities alone was centered at their true values, whereas the specificities and prevalence were assigned as non-informative priors.
3. two-step informative priors for sensitivities alone: the priors of the sensitivities were assigned according to a two-step hierarchical prior with their upper bounds assigned as much vaguer and less risky priors, as described in the *Prior distribution* section.
4. informative priors for specificities alone: the prior density of the specificities alone was centered at their true values, whereas the sensitivities and prevalence were assigned as non-informative priors.

The results of applying our models to the simulated data sets are given in Tables 6 and 7.

**Table 6:** **Posterior medians and 95 per cent posterior credible intervals for the application of the models to simulated data, with low prevalence (π = 0.01) and large sample size (n = 20000)**

True value	Informative priors for model	Informative priors for Se alone	Two-step informative priors for Se alone	Informative priors for Sp alone
π = 0.01	0.0094 (0.0079,0.0120)	0.0095 (0.0080,0.0119)	0.0093 (0.0075,0.0118)	0.0115 (0.0072,0.0590)
Se_1_ = 0.6	0.6013 (0.5035,0.6944)	0.5971 (0.5063,0.6936)	0.5972 (0.5045,0.8695)	0.4181 (0.0195,0.9603)
Se_2_ = 0.7	0.6998 (0.6047,0.7949)	0.6904 (0.6036,0.7940)	0.6816 (0.6040,0.9019)	0.3656 (0.0177,0.9636)
Sp_1_ = 0.7	0.7000 (0.6941,0.7052)	0.6998 (0.6947,0.7054)	0.700 (0.6945,0.7054)	0.6982 (0.6824,0.7057)
Sp_2_ = 0.6	0.5996 (0.5951,0.6045)	0.5999 (0.5948,0.6049)	0.5998 (0.5948,0.6048)	0.5964 (0.5762,0.6044)
pD	3.99	3.93	3.87	-14.72
DIC	53.84	53.74	53.63	35.09
π = 0.01	0.0099 (0.0089, 0.0110)	0.0099 (0.0089, 0.0110)	0.0099 (0.0090, 0.0111)	0.0133 (0.0093, 0.0377)
Se_1_ = 0.90	0.8996 (0.8526, 0.9479)	0.9019 (0.8545, 0.9479)	0.8940 (0.8528, 0.9678)	0.5017 (0.0321, 0.9563)
Se_2_ = 0.95	0.9445 (0.9020, 0.9876)	0.9429 (0.9031, 0.9875)	0.9341 (0.9015, 0.9759)	0.5130 (0.0720, 0.9736)
Sp_1_ = 0.95	0.9500 (0.9472, 0.9528)	0.9499 (0.9469, 0.9528)	0.9499 (0.9469, 0.9527)	0.9480 (0.9404, 0.9522)
Sp_2_ = 0.90	0.8998 (0.8969, 0.9027)	0.8998 (0.8969, 0.9028)	0.8997 (0.8967, 0.9027)	0.8975 (0.8906, 0.9018)
pD	3.82	4.01	4.17	-2.41
DIC	65.59	65.95	66.28	59.47

**Table 7:** **Posterior medians and 95 per cent posterior credible intervals for the application of the models to simulated data, with high prevalence (π = 0.4) and small sample size (n = 200)**

True value	Informative priors for model	Informative priors for Se alone	Two-step informative priors for Se alone	Informative priors for Sp alone
π = 0.40	0.3916 (0.3186, 0.4831)	0.3972 (0.3160, 0.5015)	0.3905 (0.3018, 0.4997)	0.3627 (0.2813, 0.5415)
Se_1_ = 0.6	0.6029 (0.5051, 0.6946)	0.6054 (0.5059, 0.6953)	0.6004 (0.5077, 0.8582)	0.6220 (0.3960, 0.9331)
Se_2_ = 0.7	0.6943 (0.6052, 0.7920)	0.6928 (0.6041, 0.7944)	0.6833 (0.6041, 0.9111)	0.7441 (0.5105, 0.9790)
Sp_1_ = 0.7	0.7009 (0.6106, 0.7915)	0.7073 (0.5872, 0.8308)	0.7093 (0.5799, 0.8478)	0.7087 (0.6046, 0.7958)
Sp_2_ = 0.6	0.5993 (0.5167, 0.6826)	0.5933 (0.5134, 0.6900)	0.5970 (0.5033, 0.7004)	0.6085 (0.5092, 0.6928)
pD	3.40	3.59	3.50	3.05
DIC	44.18	44.68	44.66	44.07
π = 0.40	0.3969 (0.3556,0.4395)	0.3971 (0.3499,0.4428)	0.3999 (0.3561,0.4462)	0.4209 (0.3588,0.6559)
Se_1_ = 0.90	0.9039 (0.8531,0.9477)	0.9011 (0.8525,0.9479)	0.8933 (0.8517,0.9581)	0.8443 (0.5371,0.9874)
Se_2_ = 0.95	0.9515 (0.9041,0.9879)	0.9531 (0.9035,0.9884)	0.9380 (0.9022,0.9808)	0.9022 (0.5775,0.9947)
Sp_1_ = 0.95	0.9549 (0.9069,0.9887)	0.9548 (0.8822,0.9968)	0.9570 (0.8863,0.9978)	0.9537 (0.9038,0.9886)
Sp_2_ = 0.90	0.9039 (0.8610,0.9415)	0.9024 (0.8459,0.9486)	0.9021(0.8496,0.9478)	0.8977 (0.8548,0.9376)
pD	2.69	3.28	3.13	0.81
DIC	29.26	30.41	30.25	27.99

Here, we would like to make the following general observations:

1. It is clear that, as long as the priors of the sensitivities were correctly set, the models with informative priors for the model, informative priors for the sensitivities alone, and two- step informative priors for the sensitivities alone produced similar results, all of which were centered over the true values. This finding suggests that the prior distributions of the prevalence and specificities could be reasonably assigned as uniform (0, 1) in these types of studies.
2. For models with informative priors for the specificities alone, the estimates of the prevalence and sensitivities were biased, and the corresponding credible intervals were very wide, suggesting that the precision of the estimated parameters was low. The negative *pD*-values meant that informative priors for the specificities alone are insufficient to estimate the parameters. Thus, the Bayesian models are non-identifiable from the perspective of probabilistic constraints [5]. The *DIC* was decreased because of the negative *pD*.
3. The parameter specificities were always estimated with greater precision than the sensitivities when the prevalence was low. Because the specificities relate to the negative subjects, when the prevalence is low, there are more truly negative subjects who provide more experimental information for specificities than for sensitivities; thus, the more precise posteriori estimates of specificities are obtained.

All of the above results indicate that prior information on the sensitivities plays a key role in these types of studies. We consider one of the reasons might be that the prevalence was low, because in absence of enough positive individuals, the prior distributions of sensitivities produced substantive influence on the posterior distribution. The results from both practical case (Tables 3 and 4) and simulation study (Tables 6 and 7) supported the consideration.

As described in the Background section, one of characteristics of two stage screening tests is that the two tests in the first stage are often conditionally dependent on disease status and/or non-disease status [5]. To further study how the prevalence and test accuracy estimates vary with the model assumptions (dependent or independent) and to evaluate the impact of the misspecification of different models on the estimation of the prevalence and test accuracy, we performed four sets of simulations under the assumptions of the independent model for independent data, the independent model for dependent data, the dependent model for independent data, and the dependent model for dependent data. For ease of presentation and interpretation, we considered moderate parameter values for the prevalence, sample size, and test accuracy, as shown below:


Table 8 shows that the independent models might generate incorrect statistical inferences for the conditional dependent data and larger *DIC* (323.08) values, indicating that the model was not appropriate for the data. For conditional independent data, the independent models and dependent models had similar results closer to their true values. Thus, we suggest that, when the researcher is uncertain as to whether the data are dependent or independent, the less risky dependent model should be used despite the slight loss of precision among the estimated parameters (widened credible interval).

**Table 8:** **Posterior medians and 95 per cent posterior credible intervals for the application of the dependent and independent models to simulated dependent and independent data (two-step informative priors for sensitivities alone were assigned (see Table** 5 **))**

	Dependent data		Independent data	
	Dependent model	Independent model	Dependent model	Independent model
*π* = 0.10	0.0963 (0.0830,0.1151)	0.1184 (0.1088,0.1277)*	0.1030 (0.0919,0.1162)	0.1000 (0.0900,0.1107)
*Se*_*1*_ = 0.70	0.6985 (0.6048,0.7960)	0.7971 (0.7841,0.7999)*	0.6558 (0.6028,0.7847)	0.6979 (0.6068,0.7940)
*Se*_*2*_ = 0.80	0.7935 (0.7044,0.8950)	0.8965 (0.8818,0.8999)*	0.7864 (0.7030,0.8942)	0.7994 (0.7057,0.8942)
*Sp*_*1*_ = 0.80	0.7978 (0.7775,0.8196)	0.8866 (0.8700,0.9028)*	0.7948 (0.7740,0.8156)	0.8002 (0.7801,0.8205)
*Sp*_*2*_ = 0.70	0.6981 (0.6792,0.7164)	0.7677 (0.7511,0.7846)*	0.6966 (0.6764,0.7170)	0.6992 (0.6797,0.7191)
pD	3.87	3.08	3.87	3.38
DIC	58.63	323.08	58.63	58.76

*: incorrect statistical inference.

## Discussion

This article presents additional methods for the estimation problem for screening studies using two screening tests with the disease statuses being verified for test positives only. The core of the methods is that Bayesian modeling is divided into two stages characterized by a joint testing strategy based on the inherent attributes of this type of screening. The first stage is a joint simultaneous testing consisting of two dependent binary tests. The second stage is a special joint sequential testing that consists of simultaneous testing in the first stage and a gold standard test in the second.

Another method presented in this study involves assigning a diffuse and less risky two-step hierarchical prior structure for sensitivities. Because reasonably informative prior information is necessary for resolving the non-identifiability problem in this type of study, we emphasized the setting of the sensitivity prior distribution based on the following three points. First, the priors of the sensitivity are more important than the priors of the specificity or prevalence when the prevalence is lower than 50%, as is often found in this type of study. Second, we placed the prior information of the sensitivity on the least possible quantity, i.e., diffuse prior or less risky prior information, such as two-step hierarchical prior structure, to reduce the influence of subjective opinion as much as possible on the results. Third, we set the prior structure to be as easy as possible for use by non-statisticians so that uniform prior distributions could be used in this study.

We found that when the priors of the sensitivities were assigned as appropriate priors, such as two-step hierarchical uniform priors, in the absence of priors for the specificities and prevalence (e.g., assigning a uniform distribution on (0,1)), the parameters involved in this study could still be estimated well. However, changing the prior structure of the sensitivities has a substantive effect on the estimators (Tables 4, 6 and 7). The reasons for these results are presented in the *Simulation studies* section. Here, we would like to state that this finding has important practical significance because the sensitivities (related to the diseased population) can often, but not always, be obtained more easily than the specificities (related to the healthy population) in medical practice. This characteristic is partially based on the increased feasibility of applying the gold standard test to the diseased population compared with the healthy population, owing to the frequent invasiveness, expense, and sophistication of many gold standard tests, particularly ones with further ethical implications. Therefore, we suggest that the prior structure of the sensitivities should be thoroughly investigated for scenarios in low-prevalence populations.

To our knowledge, this study is the first to estimate test accuracy and disease prevalence for two dependent dichotomous screening tests with unverified negative individuals on both tests using two-stage Bayesian modeling. Recently, Bohning and Patilea, Chu et al., and Li et al. proposed two-stage methods to address the non-identifiability problem [7, 8, 11]. Their two-stage estimation methods differ from ours. In the first stage, Bohning and Patilea [11], Chu et al., and Li et al. [7, 8] estimated the parameters of known cells; in the second stage, they estimated the parameters of unknown cells by making assumptions regarding the cell probabilities of various homogeneous association models. They circumvented the non-identifiability problem using a capture–recapture approach or constrained maximum likelihood estimation. Because all of the models are saturated, homogeneous association assumptions are not testable. Therefore, the dependence structure modeling considered can only be viewed as a sensitivity analysis [7]. They did suggest that Bayesian methods that incorporate prior information could be a reasonable alternative to obtain improved estimates, although further research is needed [8]. Their suggestion was an important factor in initiating our study.

Our two-stage approach has certain advantages over the previous methods. First, our method can be understood more easily and used with greater convenience by non-statisticians because the procedure of constructing the model fully agrees with the practical screening procedure. Additionally, the modeling often only involves the prior distribution for the sensitivities, because the prior distributions of the specificities and prevalence are assigned a non-informative uniform distribution of (0, 1), respectively. Moreover, the method of model checking is simple, intuitive, and convenient, as *pD* and *DIC* can be obtained directly using WinBUGS. Second, this method improves non-identifiability more reasonably, as probabilistic constraints not deterministic constraints (simplifying the model) are used. Third, the method could obtain more information to evaluate the screening strategy than the capture–recapture approach or constrained maximum likelihood estimation [7, 8, 11]. For example, the method could conveniently calculate the joint sensitivities and joint specificities and make corresponding statistical inferences, such as 95% credible intervals (Table 3). In the framework of frequency statistics, such as capture–recapture approaches or constrained maximum likelihood estimations, it is often difficult to obtain statistical inferences about the joint sensitivities and joint specificities in these types of studies.

## Conclusions

We developed a two-stage Bayesian method for two dependent dichotomous screening tests with unverified individuals who are negative on both tests. We also addressed the ad hoc model evaluation and checking procedures based on empirical information for these types of studies. Although the practical example and simulation studies considering various practical situations showed that these models fit well, three points should be clarified. (1) Due to the lack of full data, it is impossible to check the model completely without further information. (2) Although our Bayesian two-stage modeling improves non-identifiability based on probabilistic constraints, the informative prior information on the sensitivities must be assigned reasonably, even though the prior could be diffuse, as in the two-step hierarchical uniform prior. (3) Using our real example and simulated scenarios related to real-life situations, the priors of the sensitivities are more important than the priors of the specificities. However, when the prevalence is large, especially if it is greater than 50% (rare in medical screening tests), the same attention should be paid to the priors of the specificities.

## Electronic supplementary material

Additional file 1: **The WinBUGS code, data and results.** (DOC 40 KB)

Additional file 2: Figure S1: The convergence diagnosis. (PDF 972 KB)

Additional file 3: **The technical details of model evaluation and checking.** (DOCX 21 KB)

## Abbreviations

BCI: Bayesian credible interval
DIC: Deviance Information Criterion
pD: Effective number of parameters
PPV: Positive predictive value
SI: Simultaneous testing.
