# Supplementary material for: A two-stage Bayesian method for estimating accuracy and disease prevalence for two dependent dichotomous screening tests when the status of individuals who are negative on both tests is unverified
Source: BMC Med Res Methodol. 2014 Sep 23;14:110. doi: 10.1186/1471-2288-14-110 (PMC4193534; doi:10.1186/1471-2288-14-110)
Supplement: Supplementary file 1 — Additional file 1: The WinBUGS code, data and results. (DOC 40 KB) [file 12874_2014_1125_MOESM1_ESM.doc]

**File name: Additional file 1:** The WinBUGS code, data and results.

Author: Dr. Jin Liu

Department of Epidemiology and Biostatistics,

School of Public Health, Nanjing Medical University

Date: July, 2014

WinBUGS 1.4 code to accompany the manuscript entitled "A two-stage Bayesian method to estimate the accuracy and disease prevalence for two dependent dichotomous screening tests when the status of individuals negative on both tests is unverified” submitted to *BMC Medical Research Methodology*. July 7, 2014

The data from Table 2 and the detailed results for Table 3.

***********************************************************************************************************************************

model

{

#First-Stage model

x[1:4] ~ dmulti(p[1:4], n) # likelihood of having observed data 'x'

p[1] <- pi*(Se1*Se2+covDp) + (1-pi)*((1-Sp1)*(1-Sp2)+covDn)

p[2] <- pi*(Se1*(1-Se2)-covDp) + (1-pi)*((1-Sp1)*Sp2-covDn)

p[3] <- pi*((1-Se1)*Se2-covDp) + (1-pi)*(Sp1*(1-Sp2)-covDn)

p[4] <- pi*((1-Se1)*(1-Se2)+covDp) + (1-pi)*(Sp1*Sp2+covDn)

covDp ~ dunif(0, SeUB)

covDn ~ dunif(0, SpUB)

SeUB<- min(Se1,Se2) - Se1*Se2

SpUB<- min(Sp1,Sp2) - Sp1*Sp2

rhoDp<- covDp / sqrt(Se1*(1-Se1)*Se2*(1-Se2))

rhoDn <- covDn / sqrt(Sp1*(1-Sp1)*Sp2*(1-Sp2))

Seje<-Se1+Se2-Se1*Se2-covDp

Spje<-Sp1*Sp2+covDn

#Second-stage model

a[1:3]~dmulti(p1[1:3],n) # likelihood of having observed data 'a'

p1[1]<-pi*Seje

p1[2]<-(1-pi)*(1-Spje)

p1[3]<-1-p1[1]-p1[2]

PPVje<-Seje*pi/(Seje*pi+(1-Spje)*(1-pi))

# priors

Se1~dunif(0.5,b1) #Firdt step prior for Sei

Se2~dunif(0.6,b2)

b1~dunif(0.55,0.99) #Second step prior for Sei

b2~dunif(0.65,0.99)

Sp1~dunif(0,1) #uniform (0,1) proir for Spi and pi

Sp2~dunif(0,1)

pi~dunif(0,1)

}

# INIT

list(pi=0.005,Se1=0.8, Sp1=0.6,Se2=0.8, Sp2=0.6)

list(pi=0.1,Se1=0.6,Sp1=0.4,Se2=0.6, Sp2=0.4)

# DATA

list(n=5727,x=c(39,91,237,5360),a=c(29,338,5360))

Node statistics

| **node** | **mean** | **sd** | **MC error** | **2.5%** | **median** | **97.5%** | **start** | **sample** |
| --- | --- | --- | --- | --- | --- | --- | --- | --- |
| PPVje | 0.08139 | 0.01397 | 4.246E-5 | 0.05599 | 0.08072 | 0.1106 | 5000 | 105000 |
| Se1 | 0.6314 | 0.1046 | 5.416E-4 | 0.5048 | 0.604 | 0.8834 | 5000 | 105000 |
| Se2 | 0.7041 | 0.08243 | 4.245E-4 | 0.6039 | 0.6825 | 0.9036 | 5000 | 105000 |
| Seje | 0.8077 | 0.08177 | 4.291E-4 | 0.6503 | 0.8103 | 0.9557 | 5000 | 105000 |
| Sp1 | 0.9811 | 0.00194 | 6.892E-6 | 0.9772 | 0.9812 | 0.9849 | 5000 | 105000 |
| Sp2 | 0.9558 | 0.002295 | 6.52E-6 | 0.9513 | 0.9559 | 0.9603 | 5000 | 105000 |
| Spje | 0.9405 | 0.00229 | 5.474E-6 | 0.936 | 0.9405 | 0.945 | 5000 | 105000 |
| pi | 0.006547 | 0.001325 | 5.501E-6 | 0.004254 | 0.006441 | 0.009433 | 5000 | 105000 |
