# Supplementary figures and images for: A two-stage Bayesian method for estimating accuracy and disease prevalence for two dependent dichotomous screening tests when the status of individuals who are negative on both tests is unverified
Source: BMC Med Res Methodol. 2014 Sep 23;14:110. doi: 10.1186/1471-2288-14-110 (PMC4193534; doi:10.1186/1471-2288-14-110)

# Gelman Rubin statistic

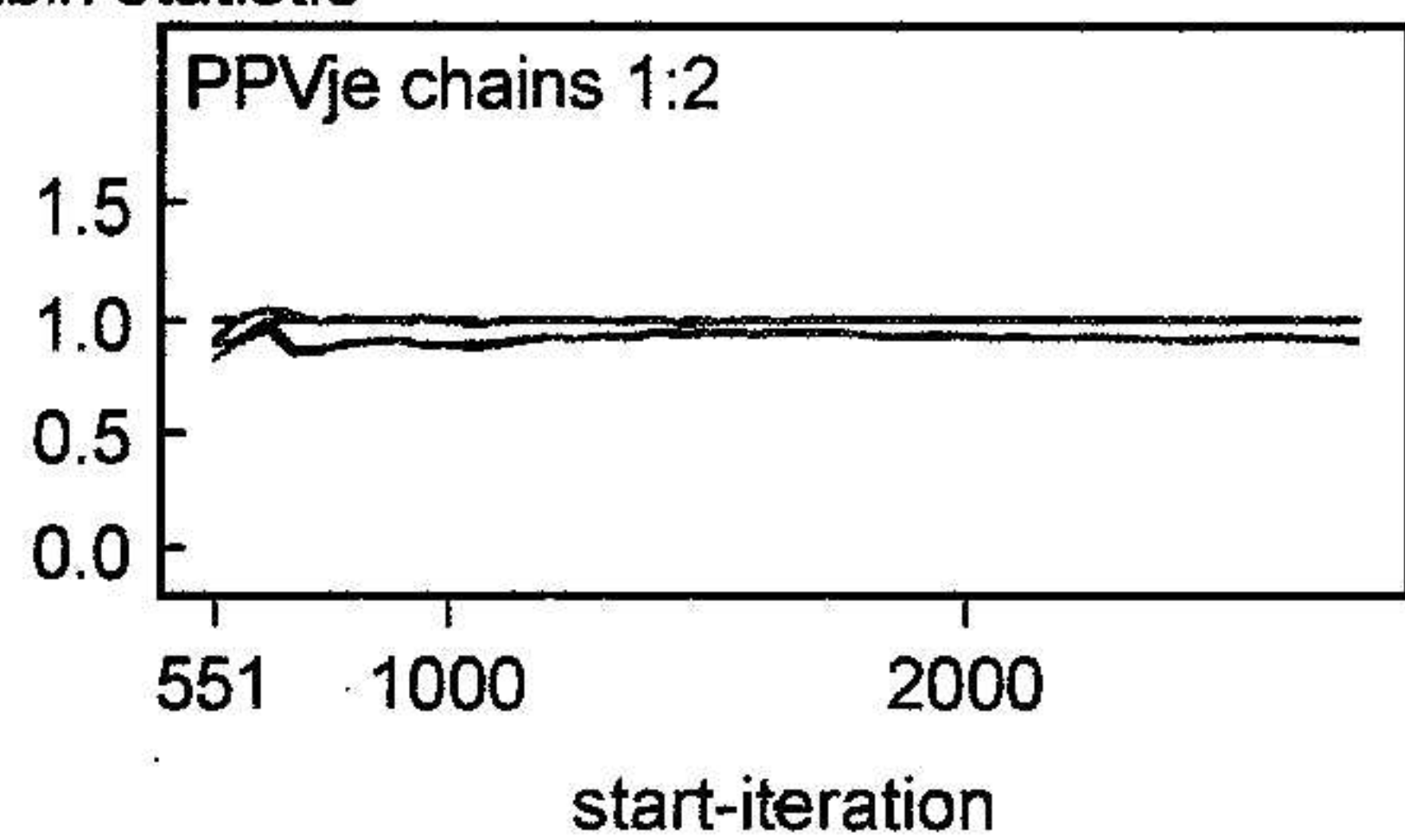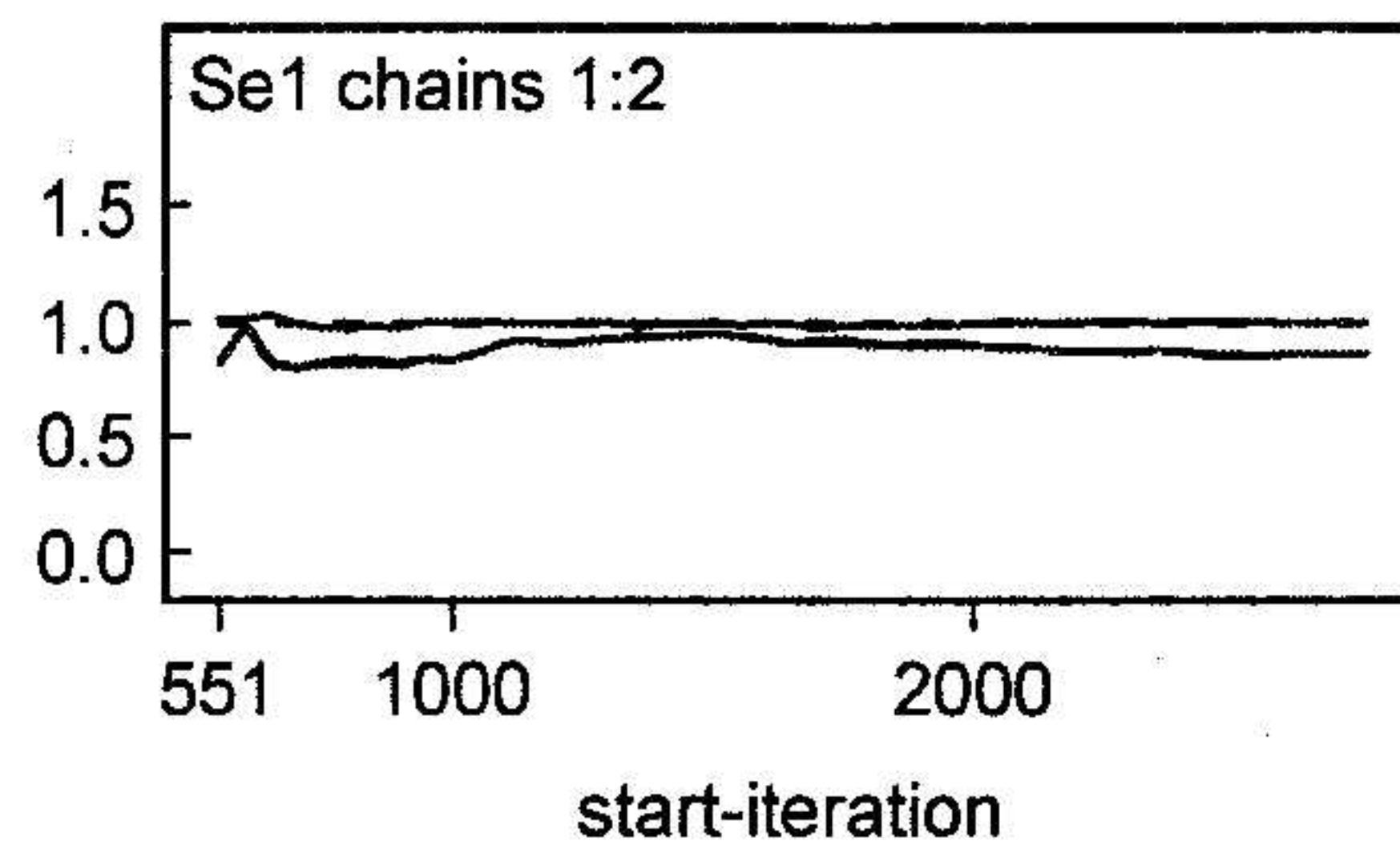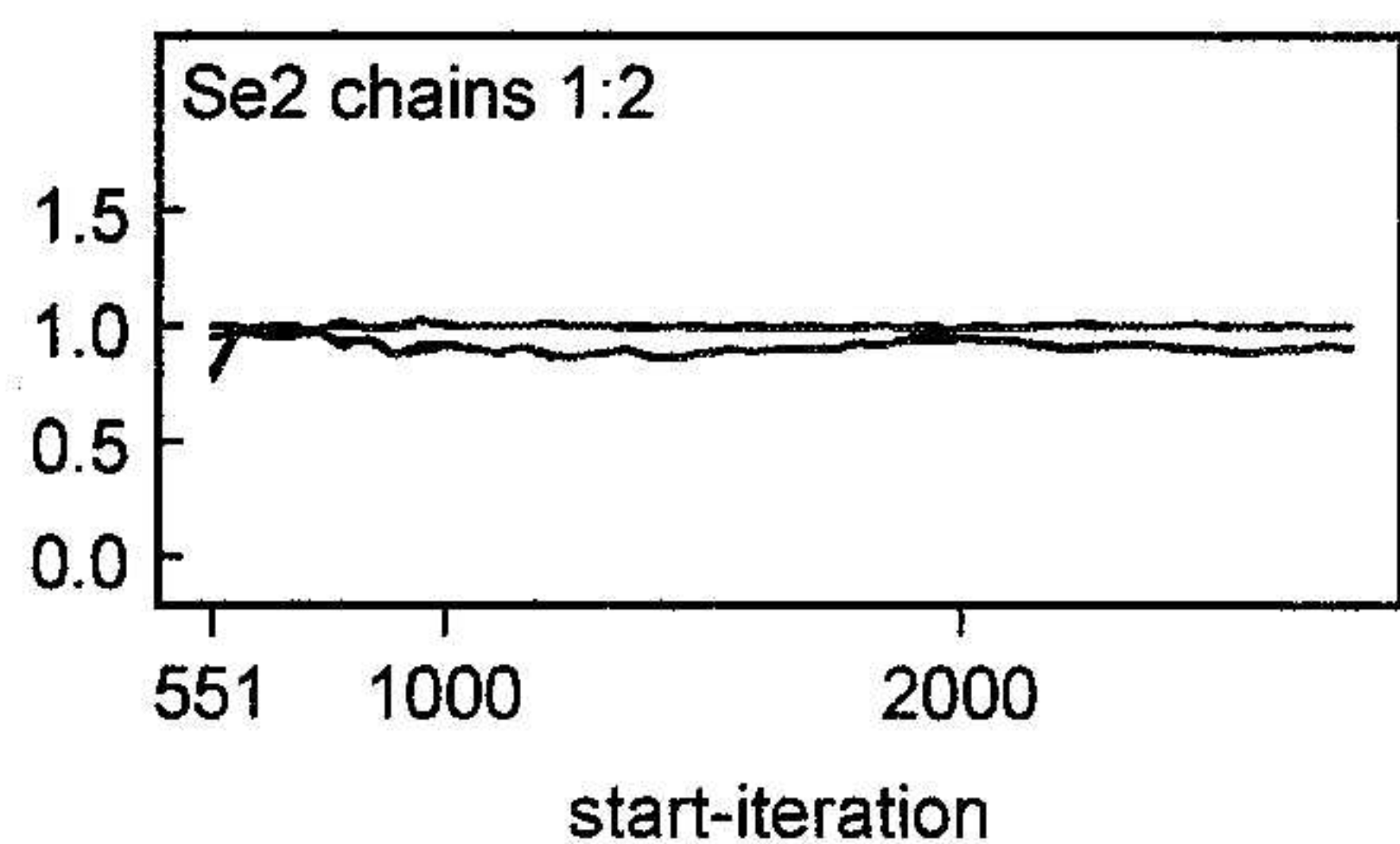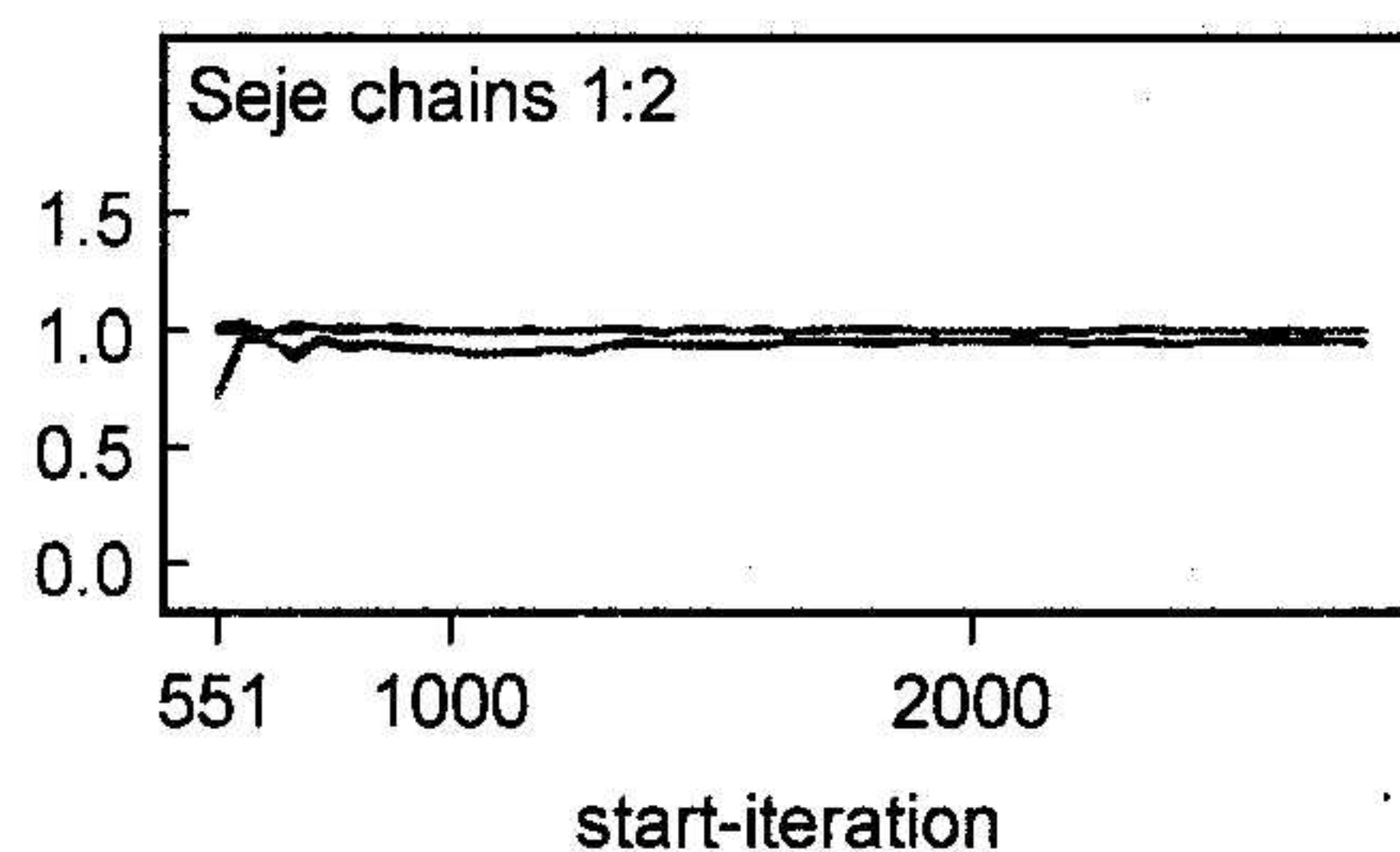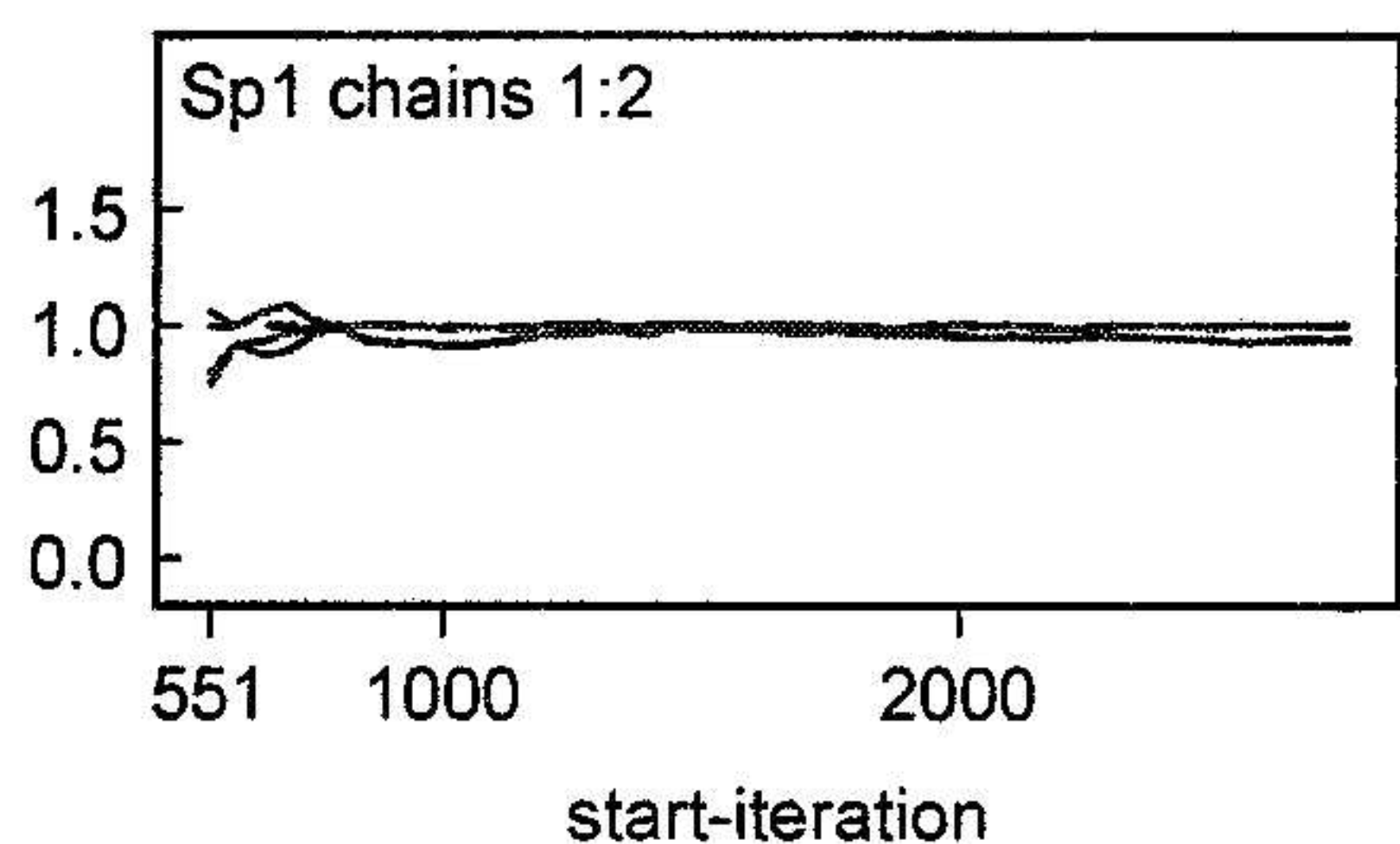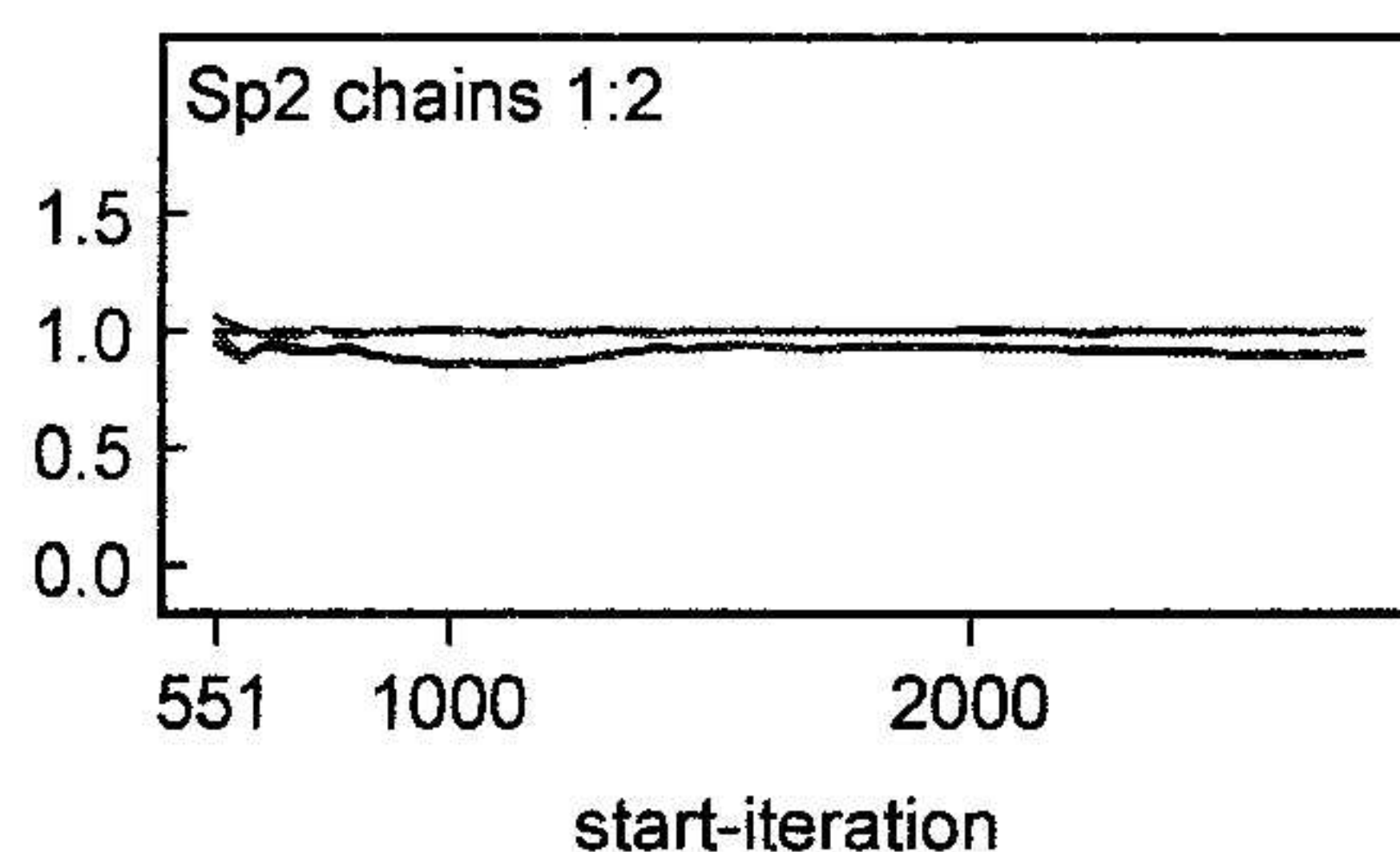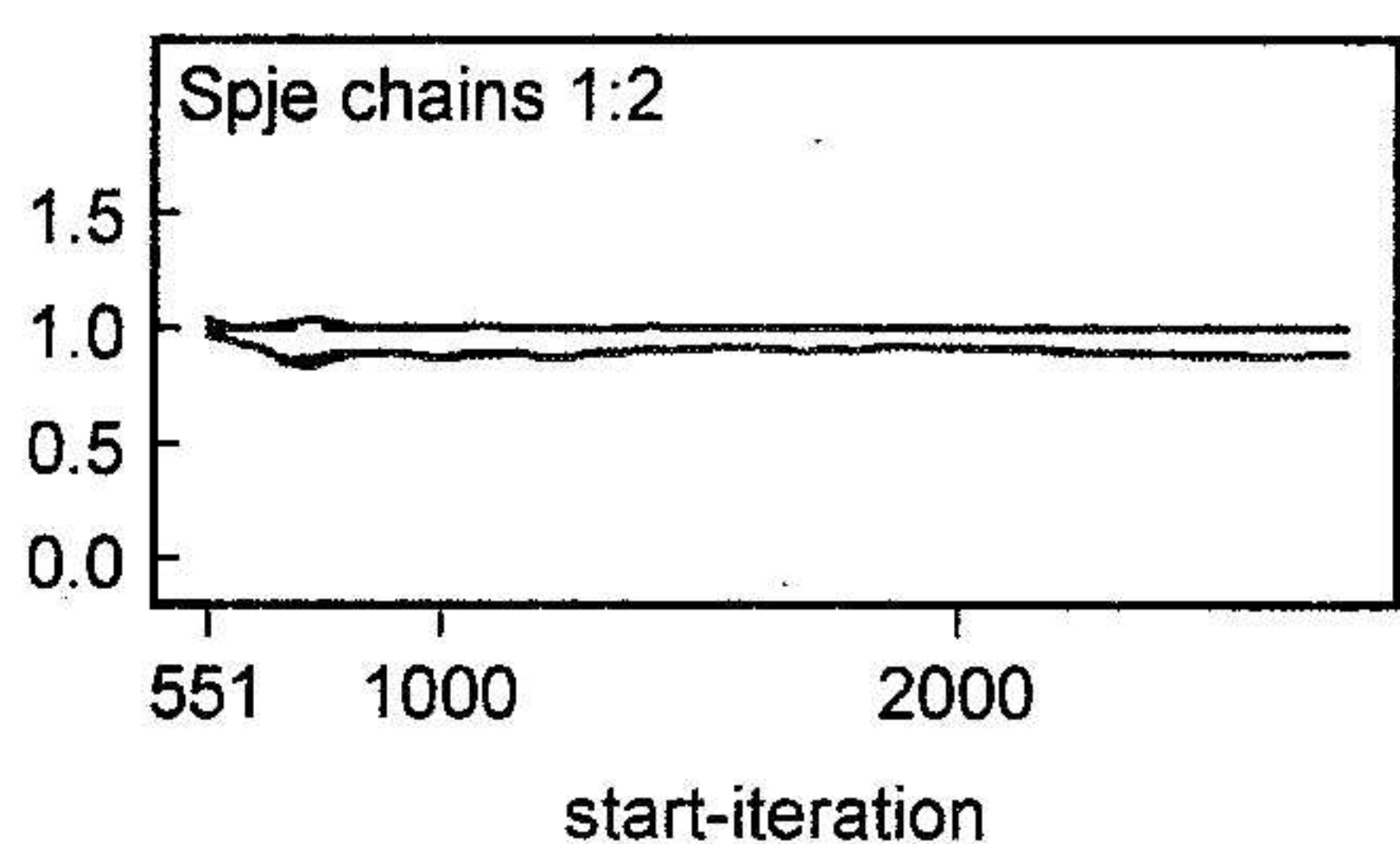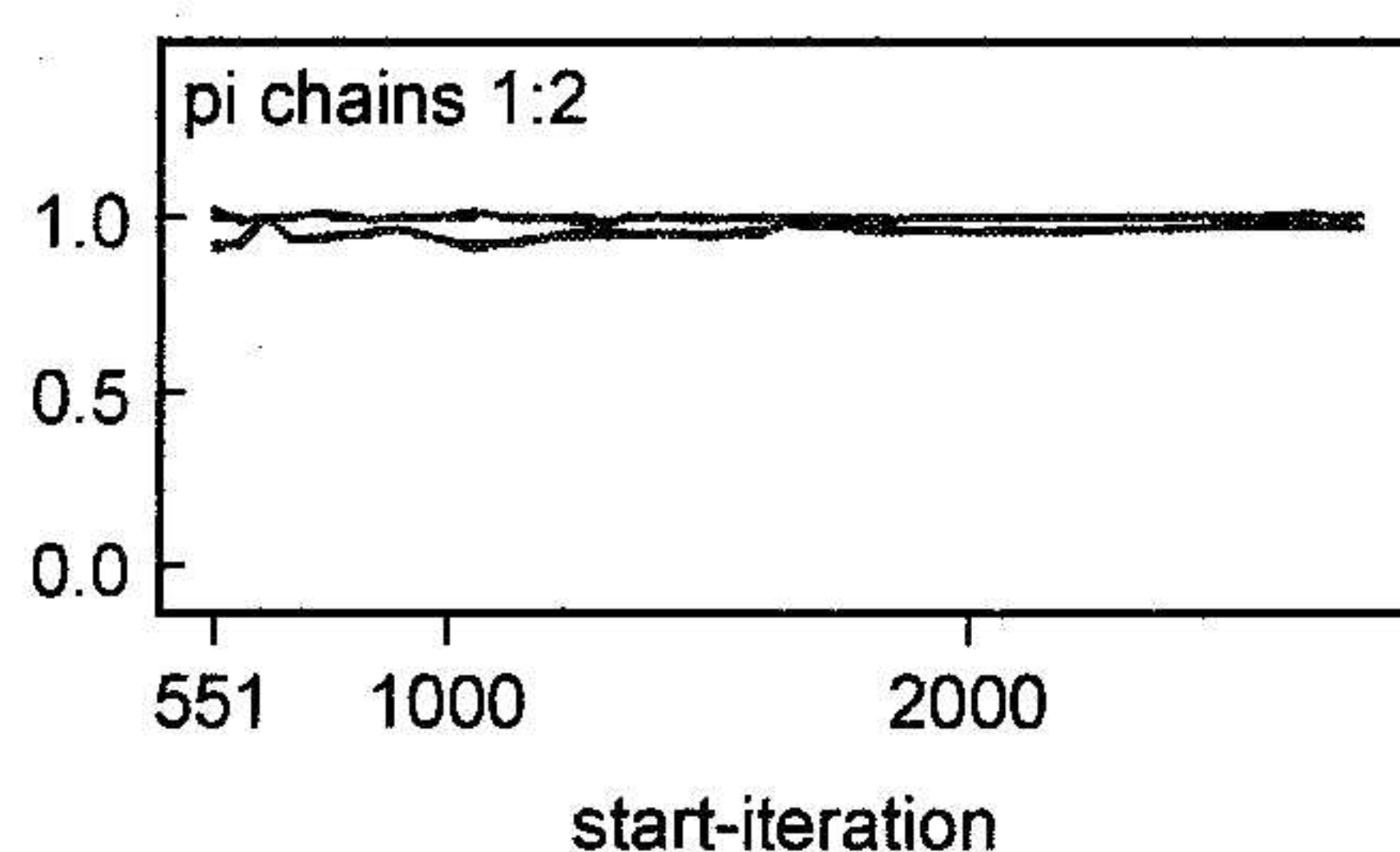

## Running quantiles

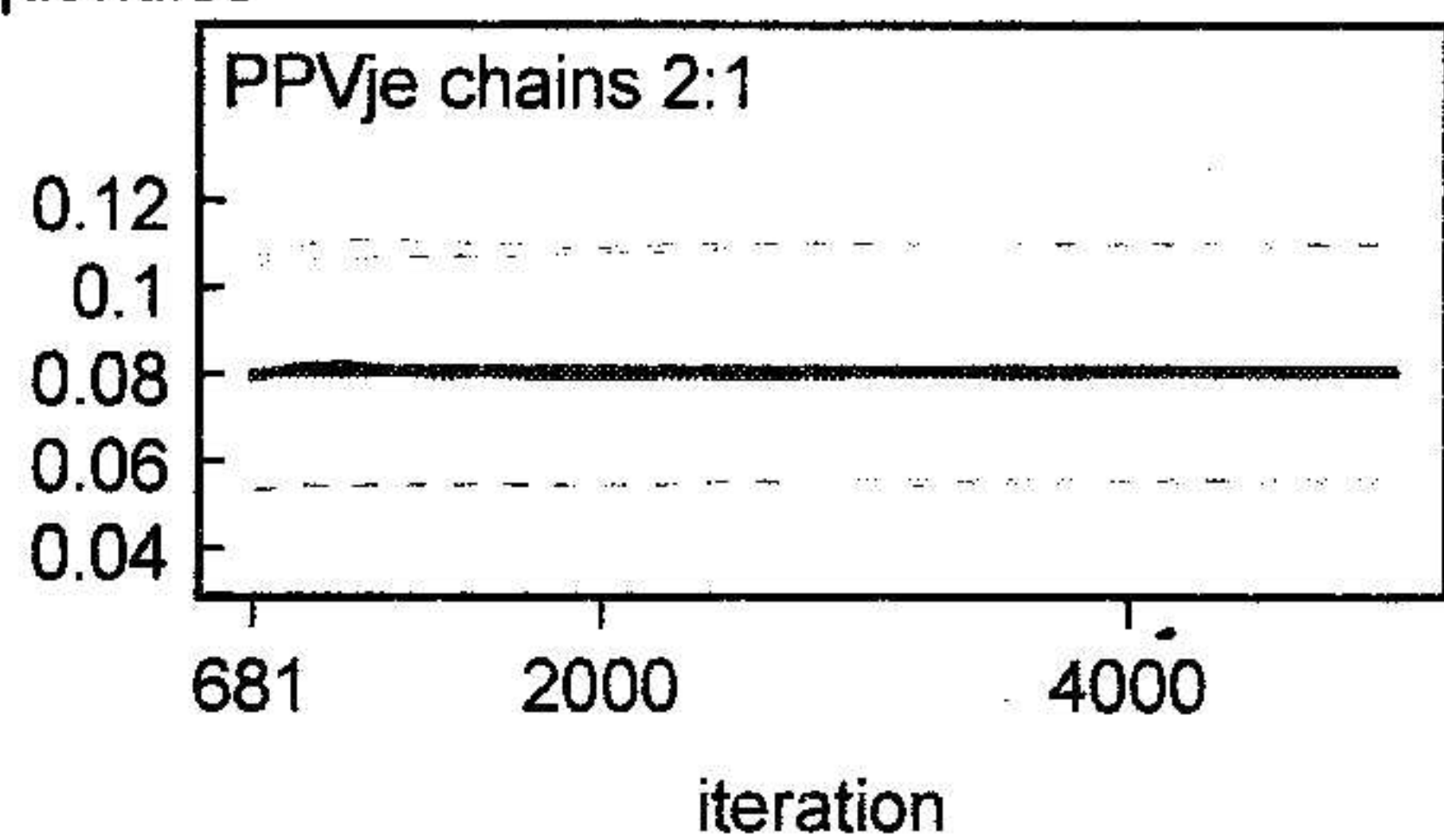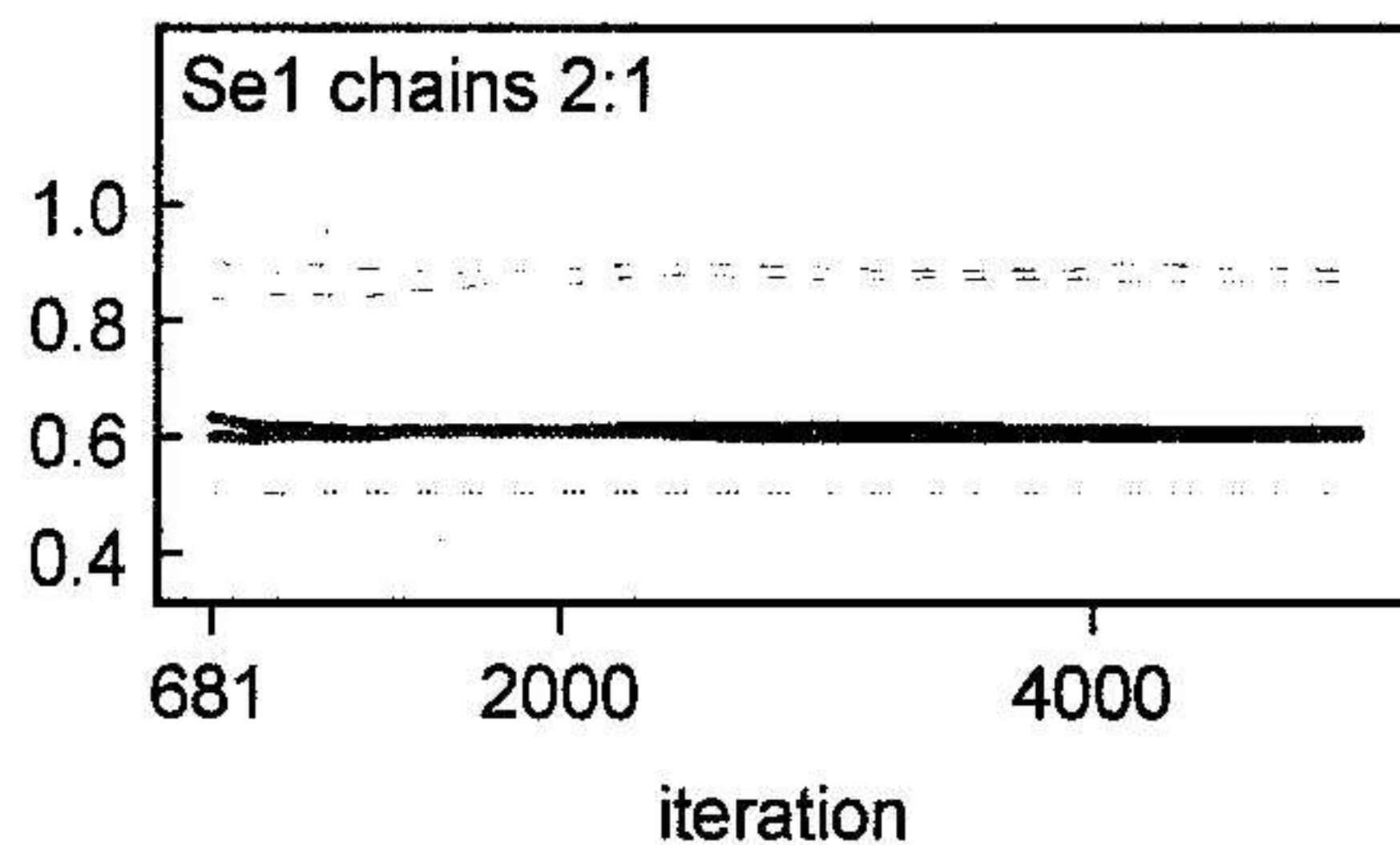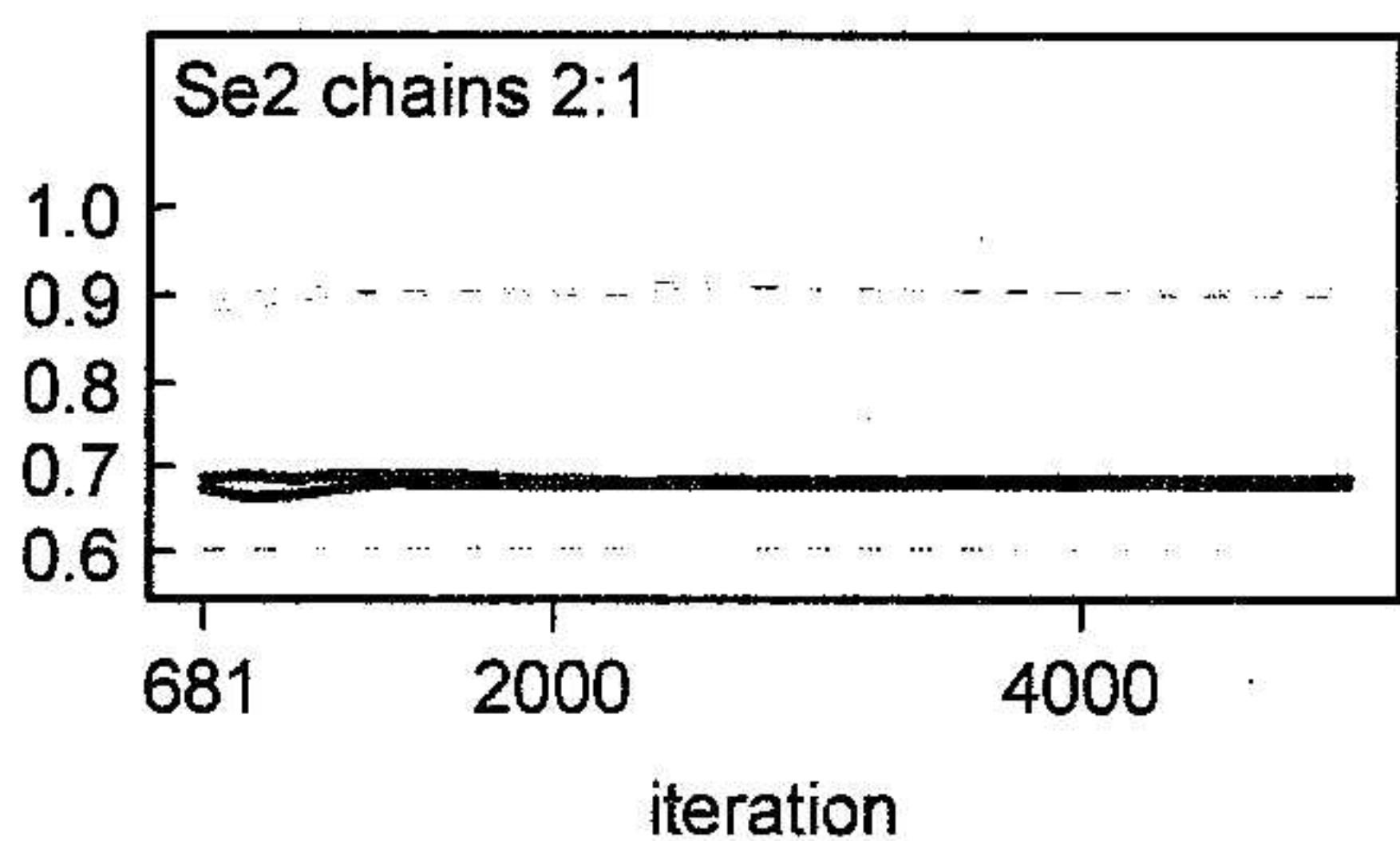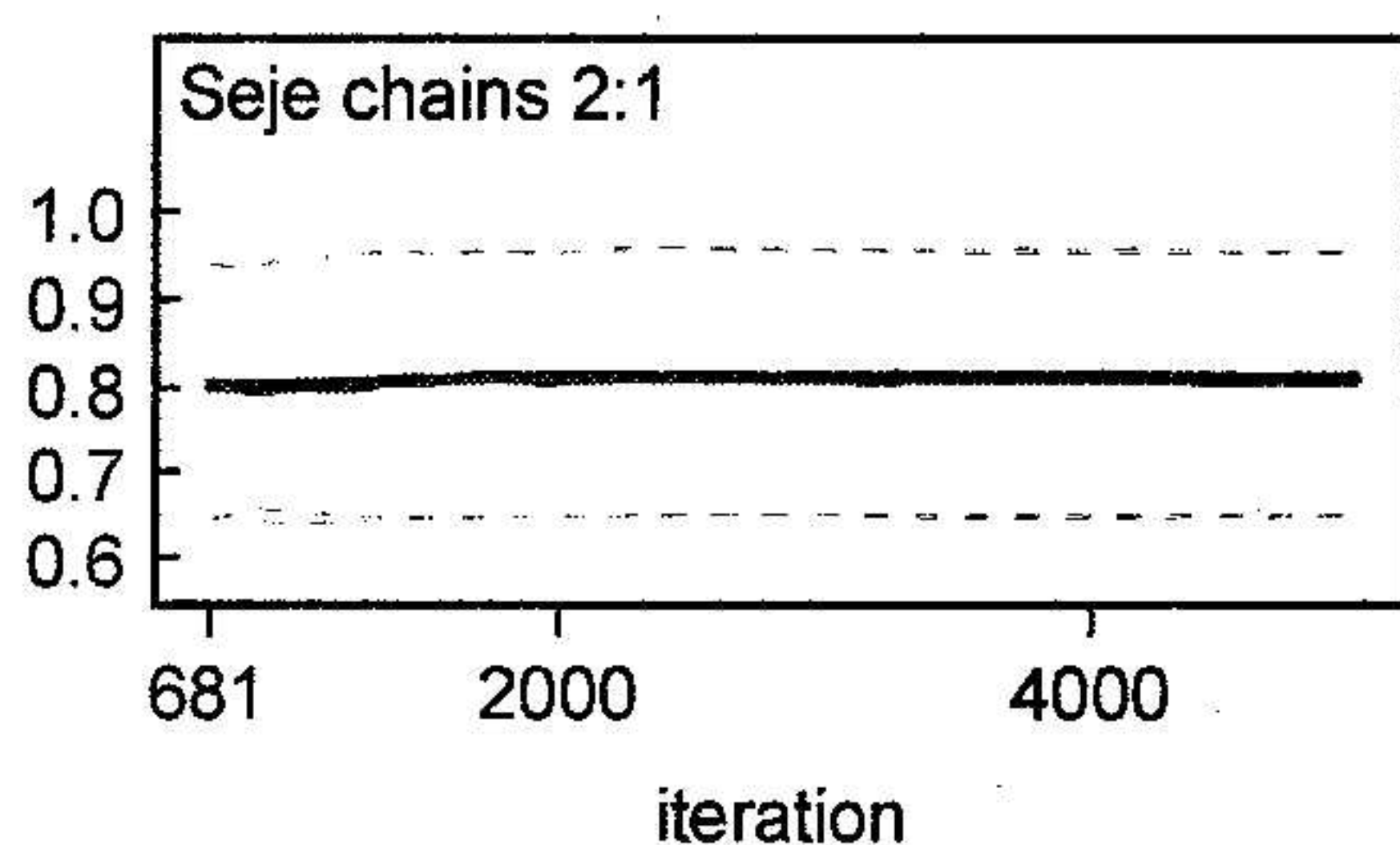

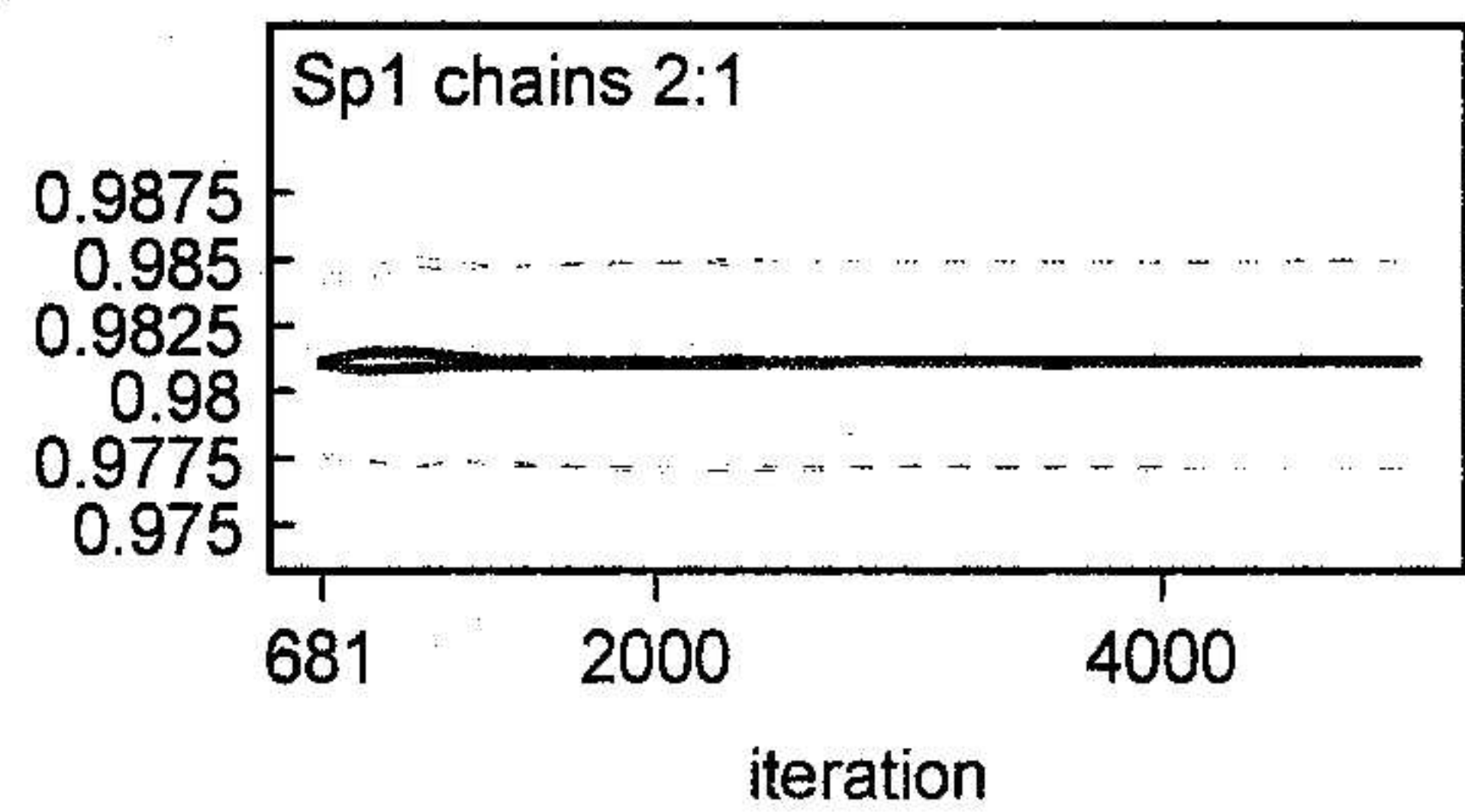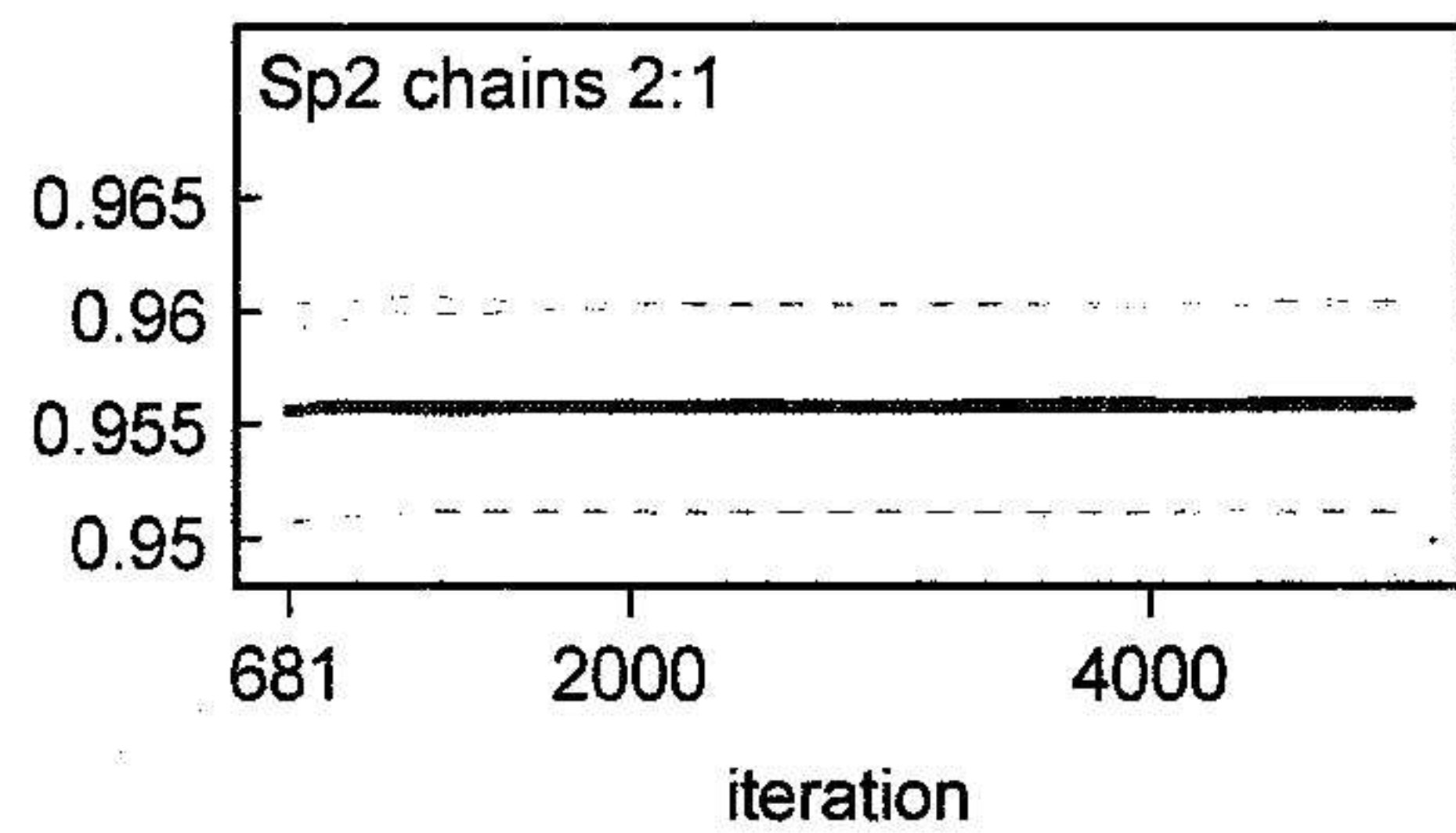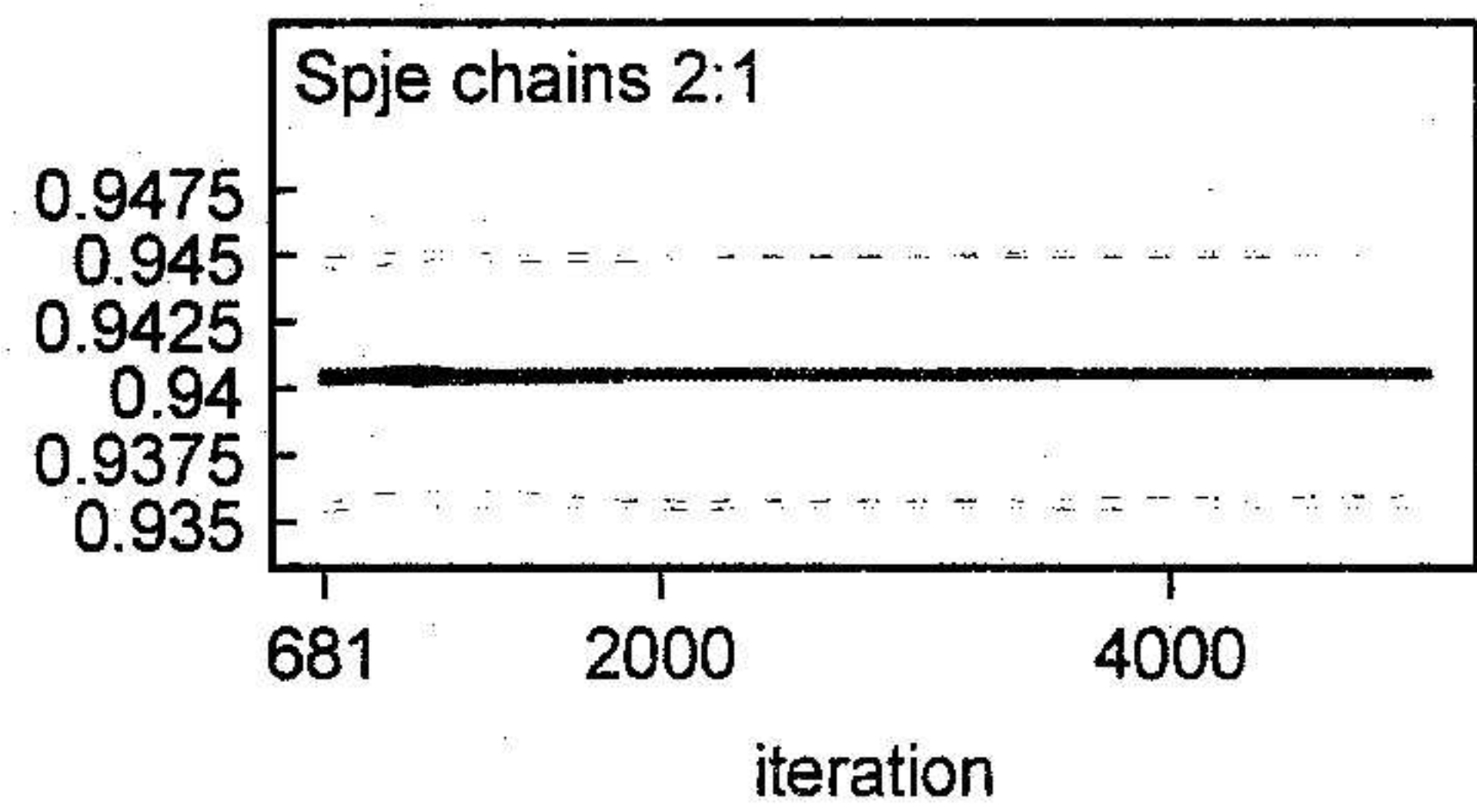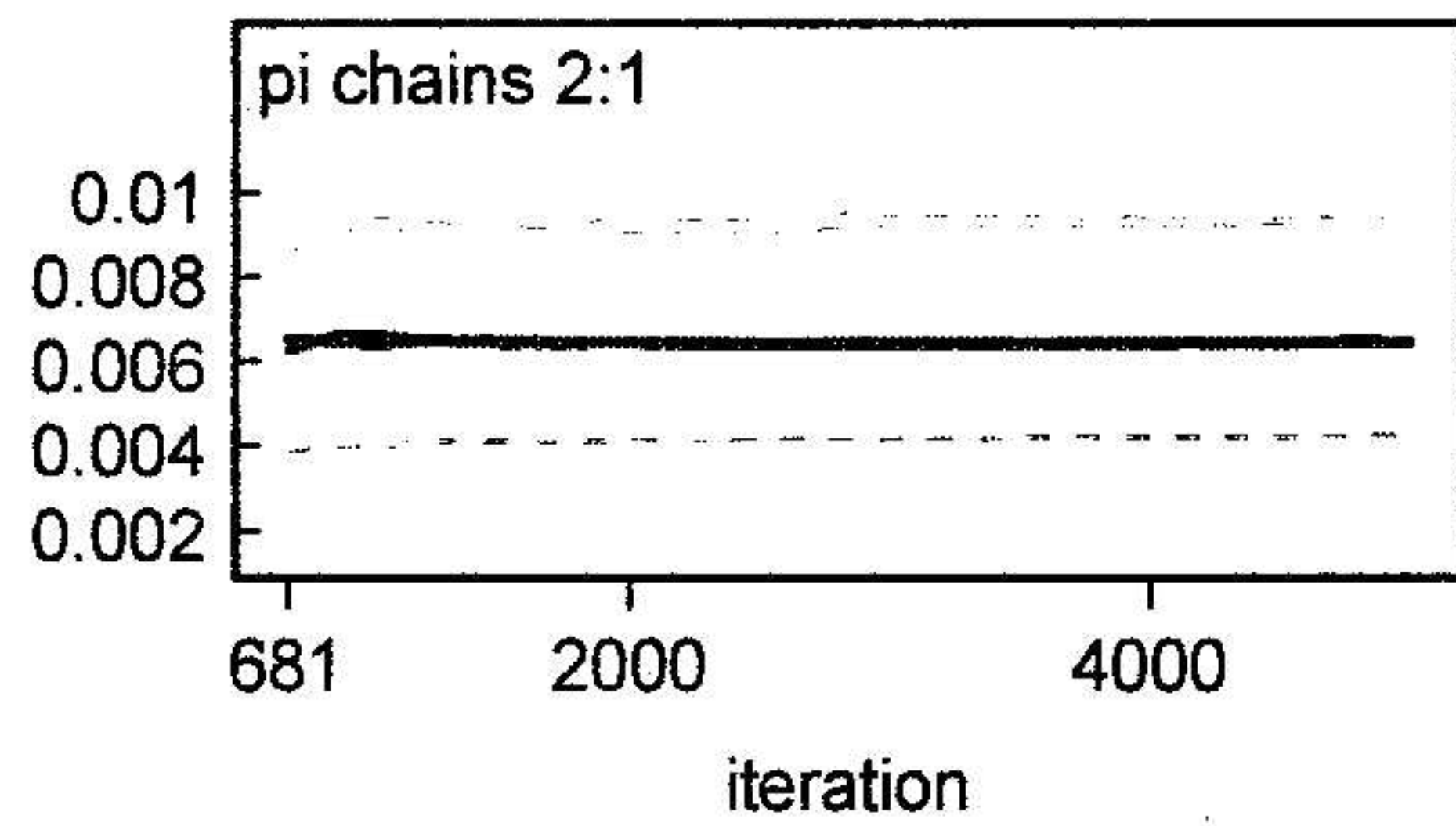

Dynamic trace

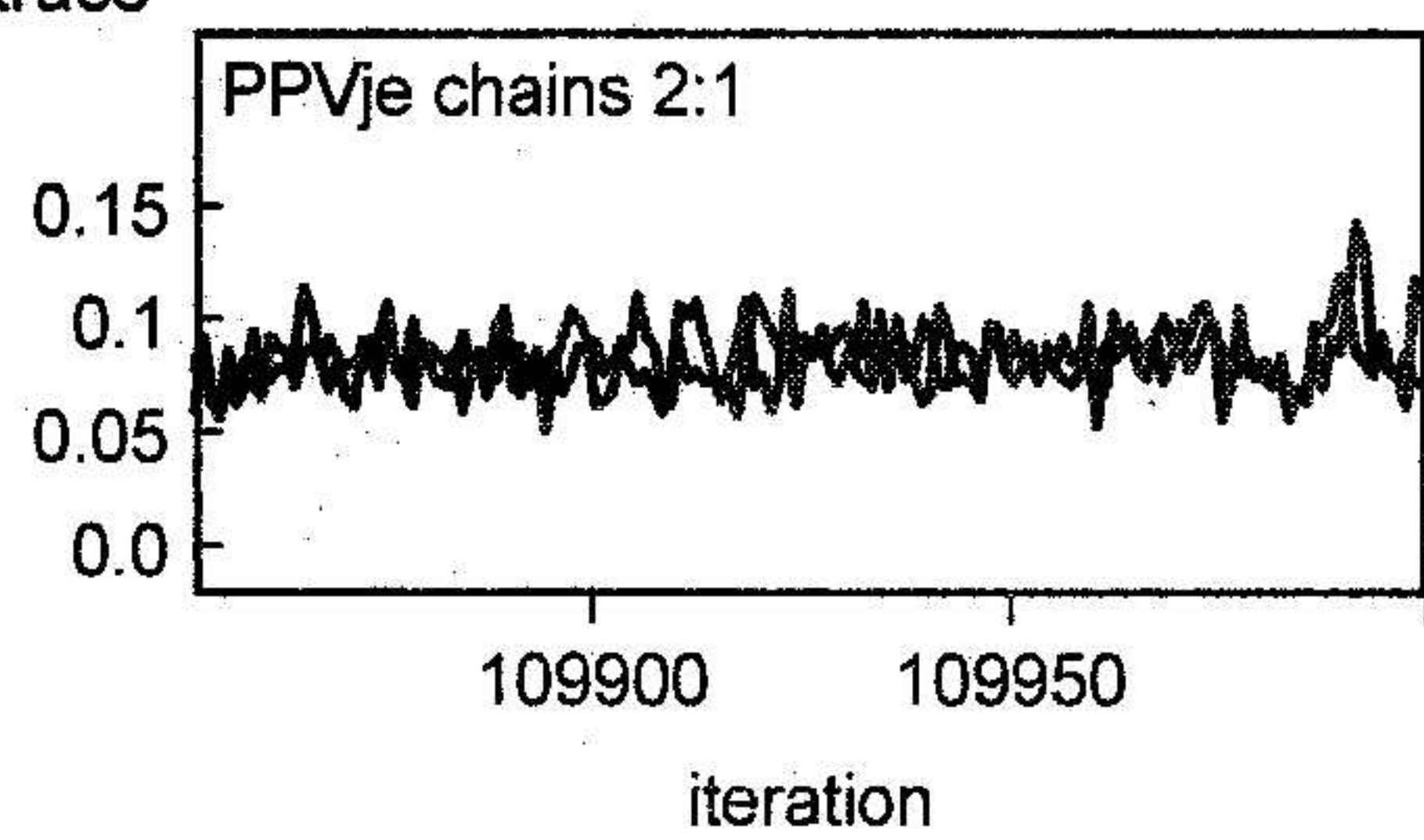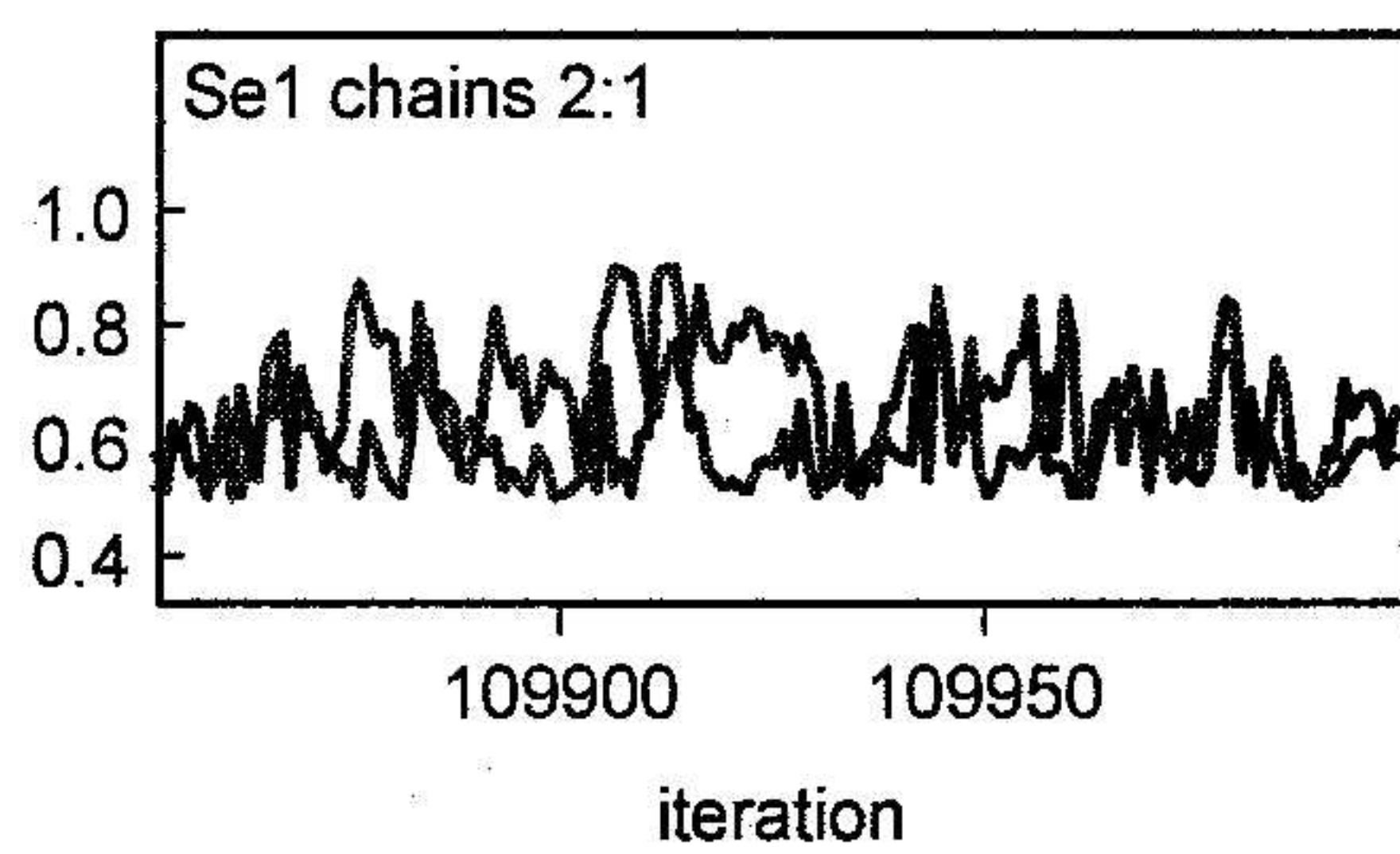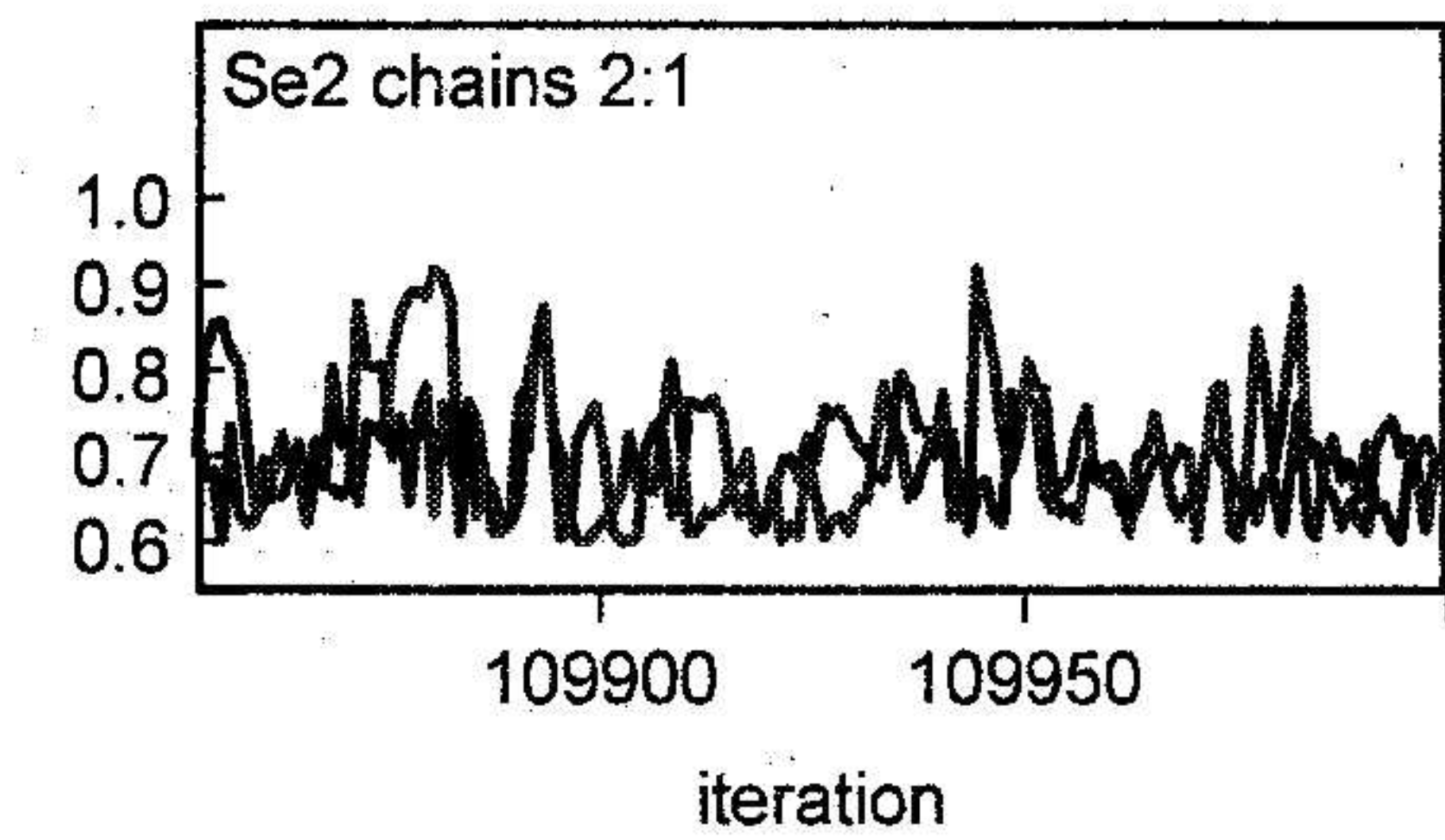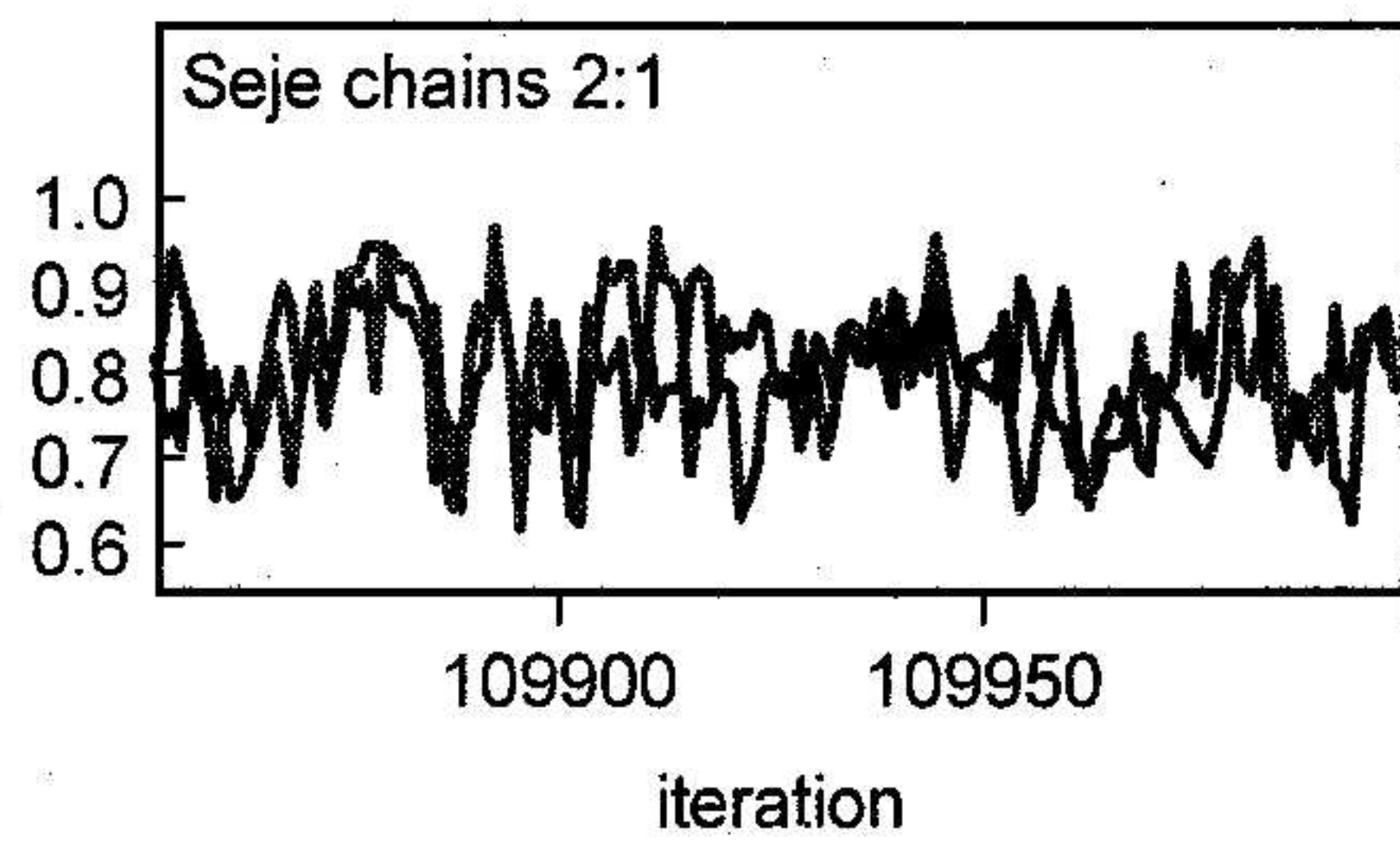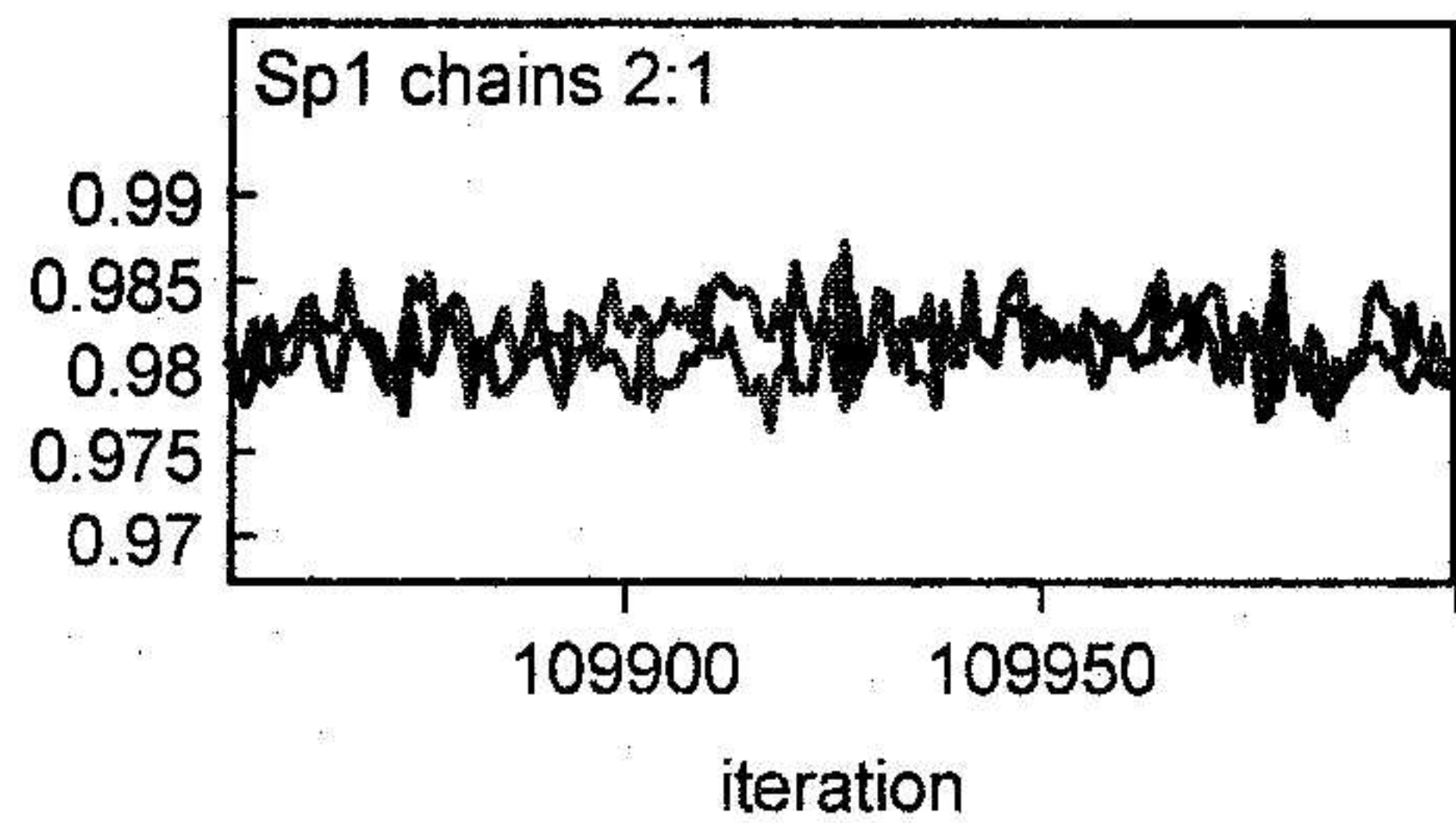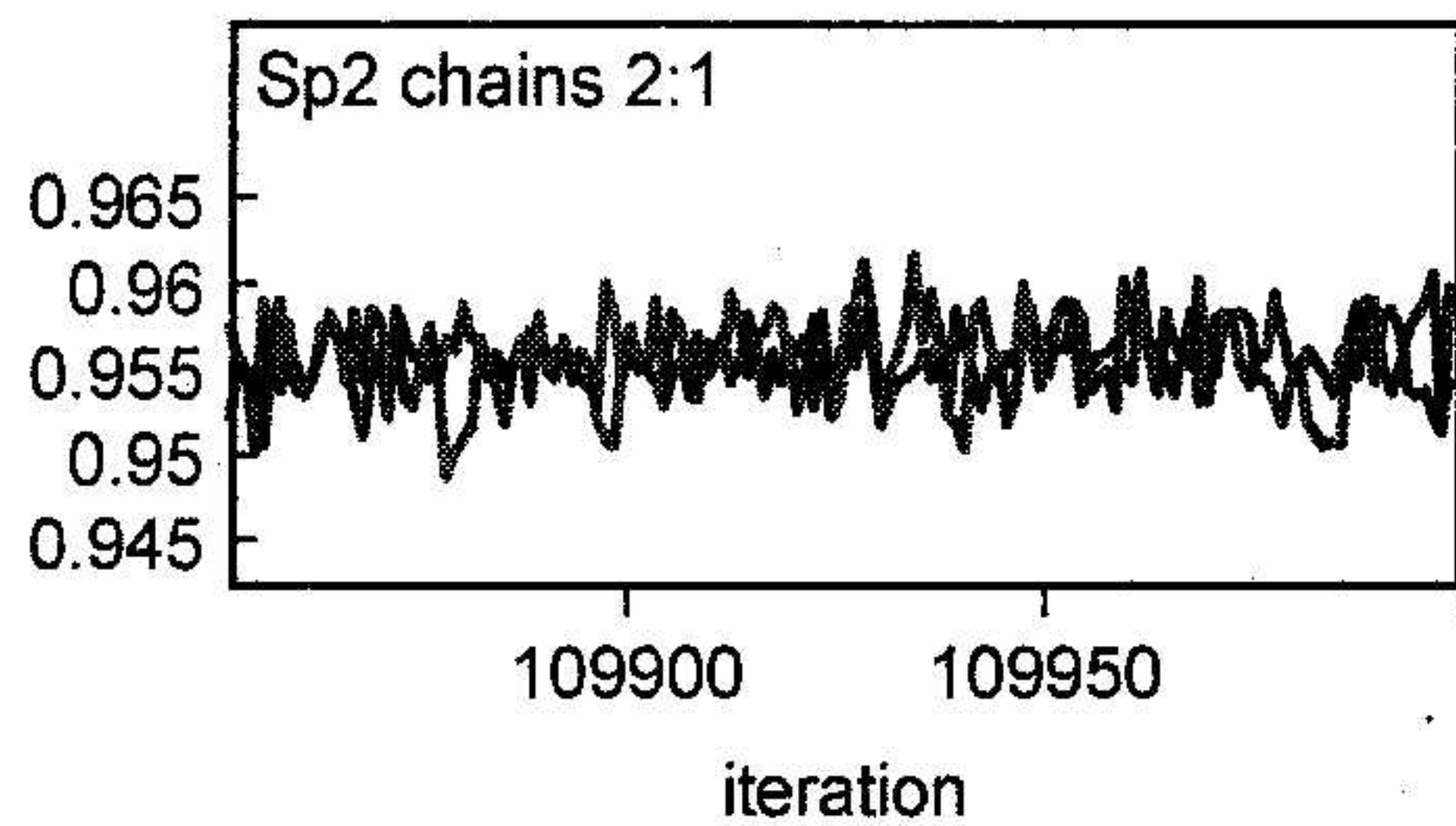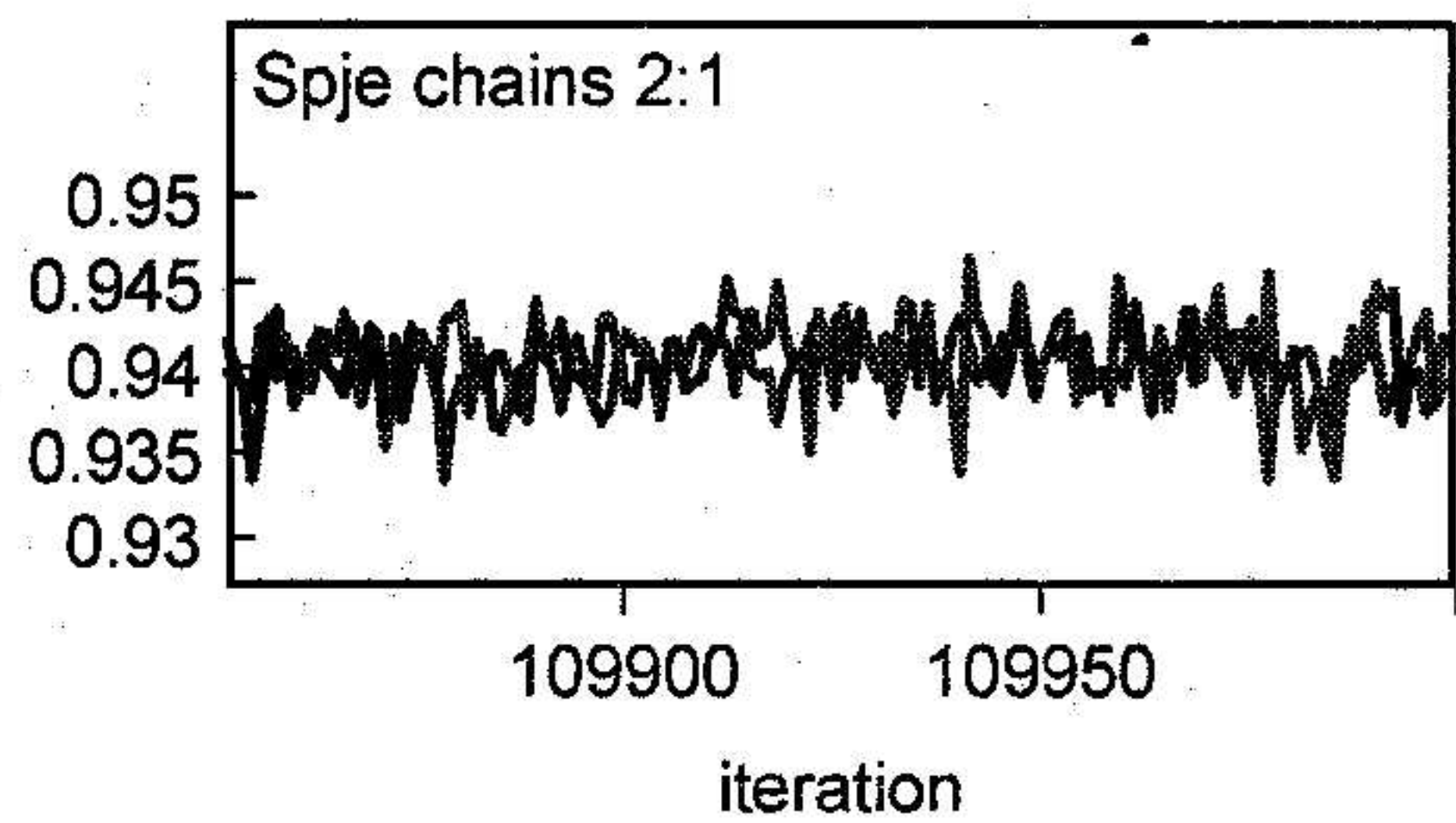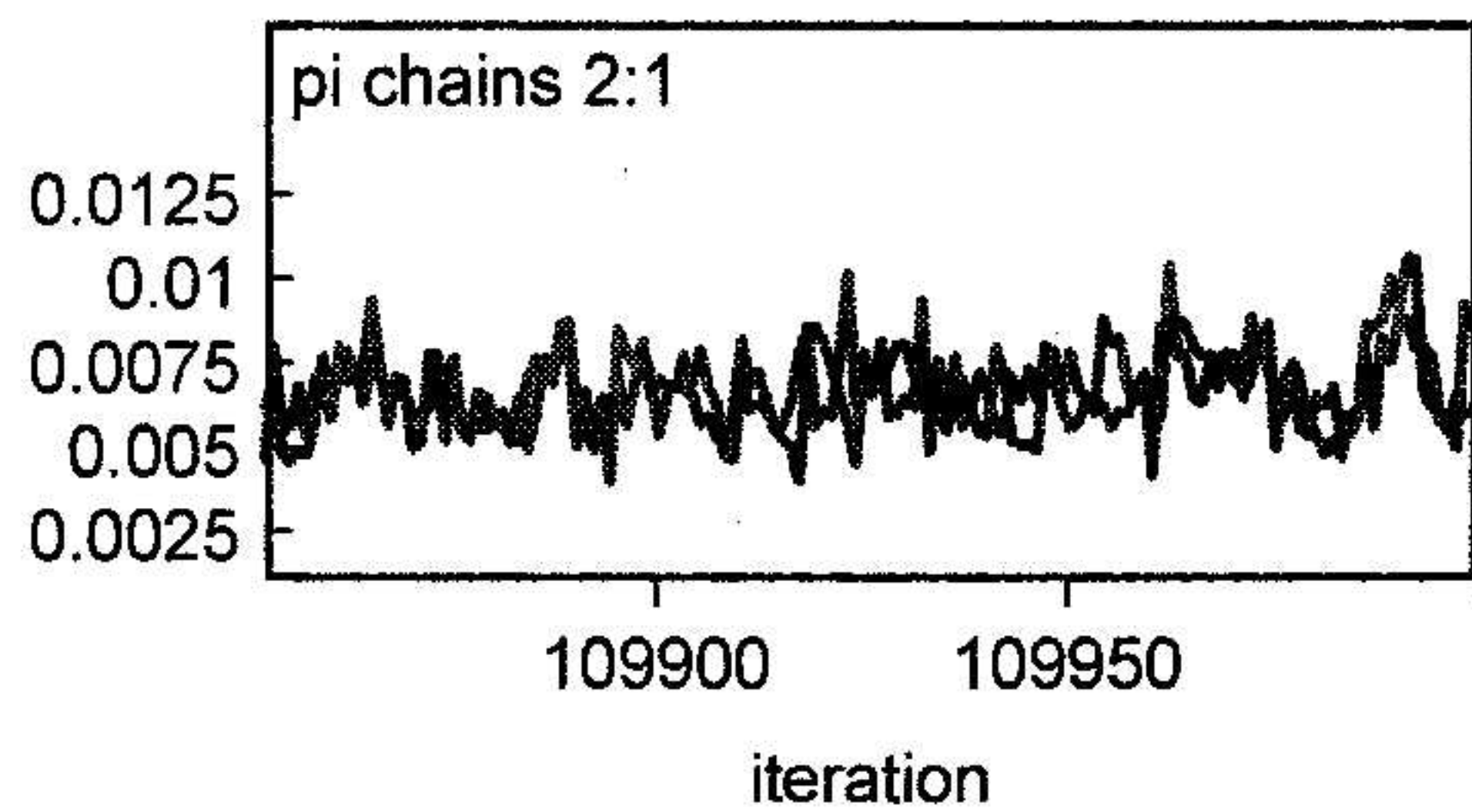

Autocorrelation function

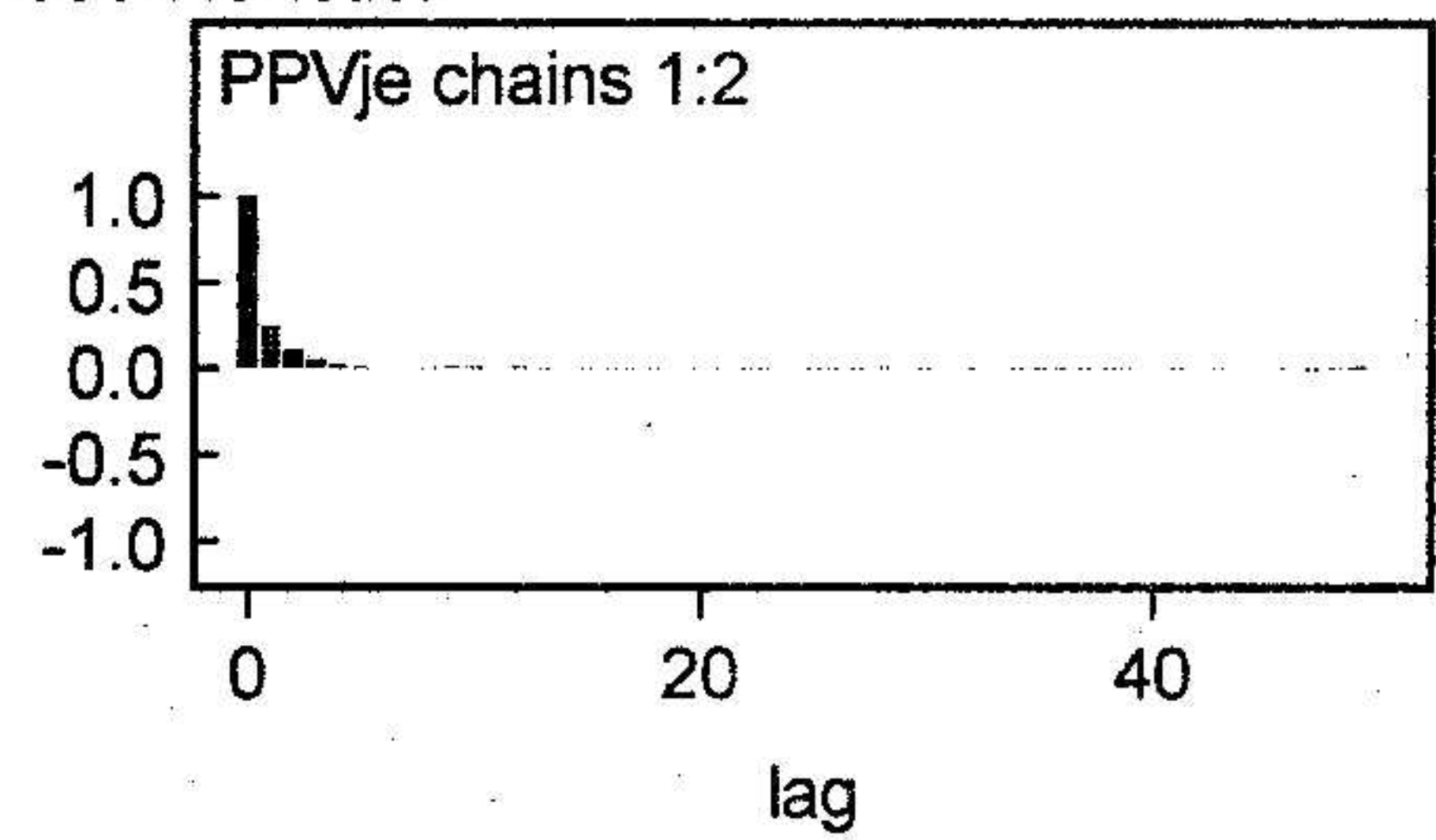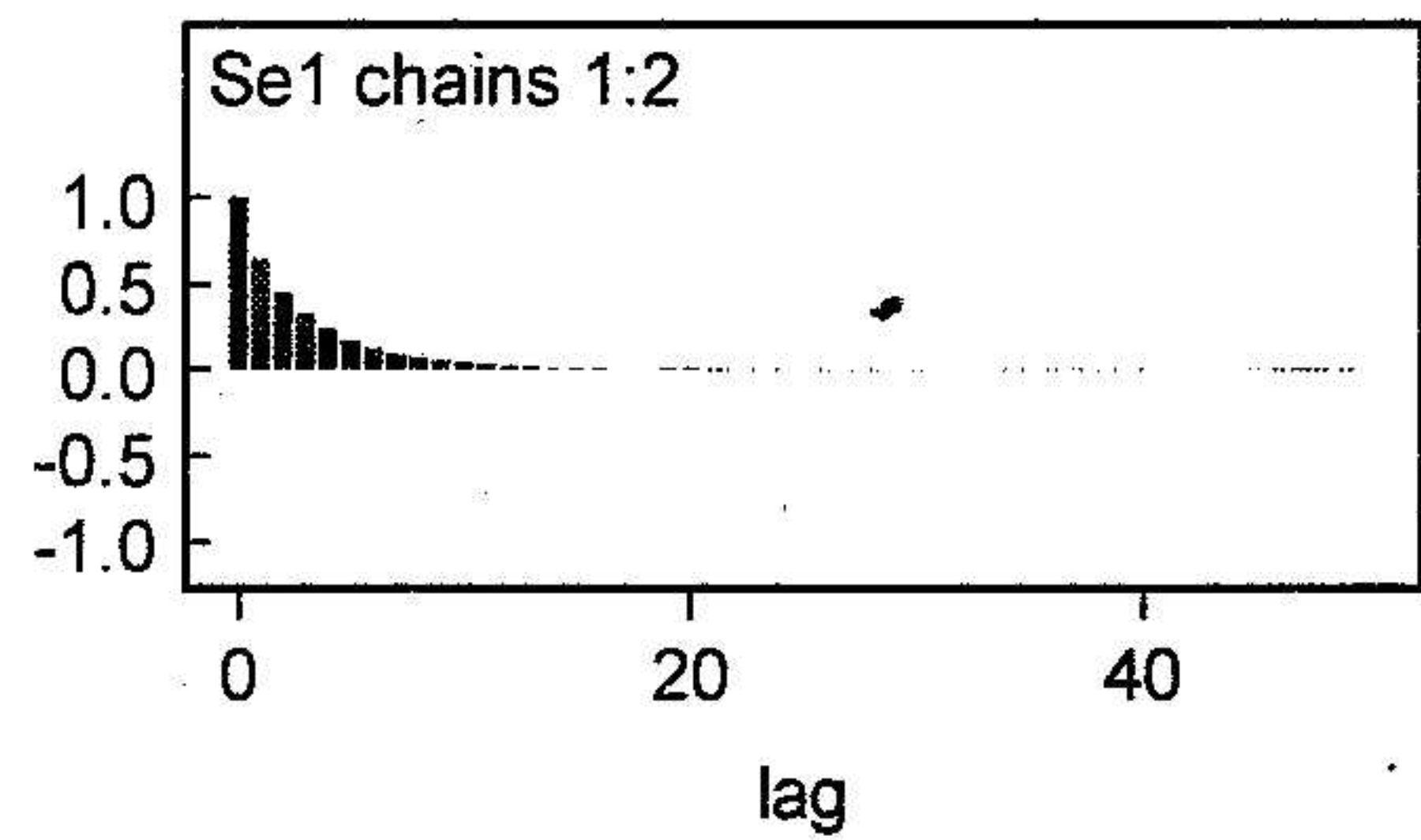

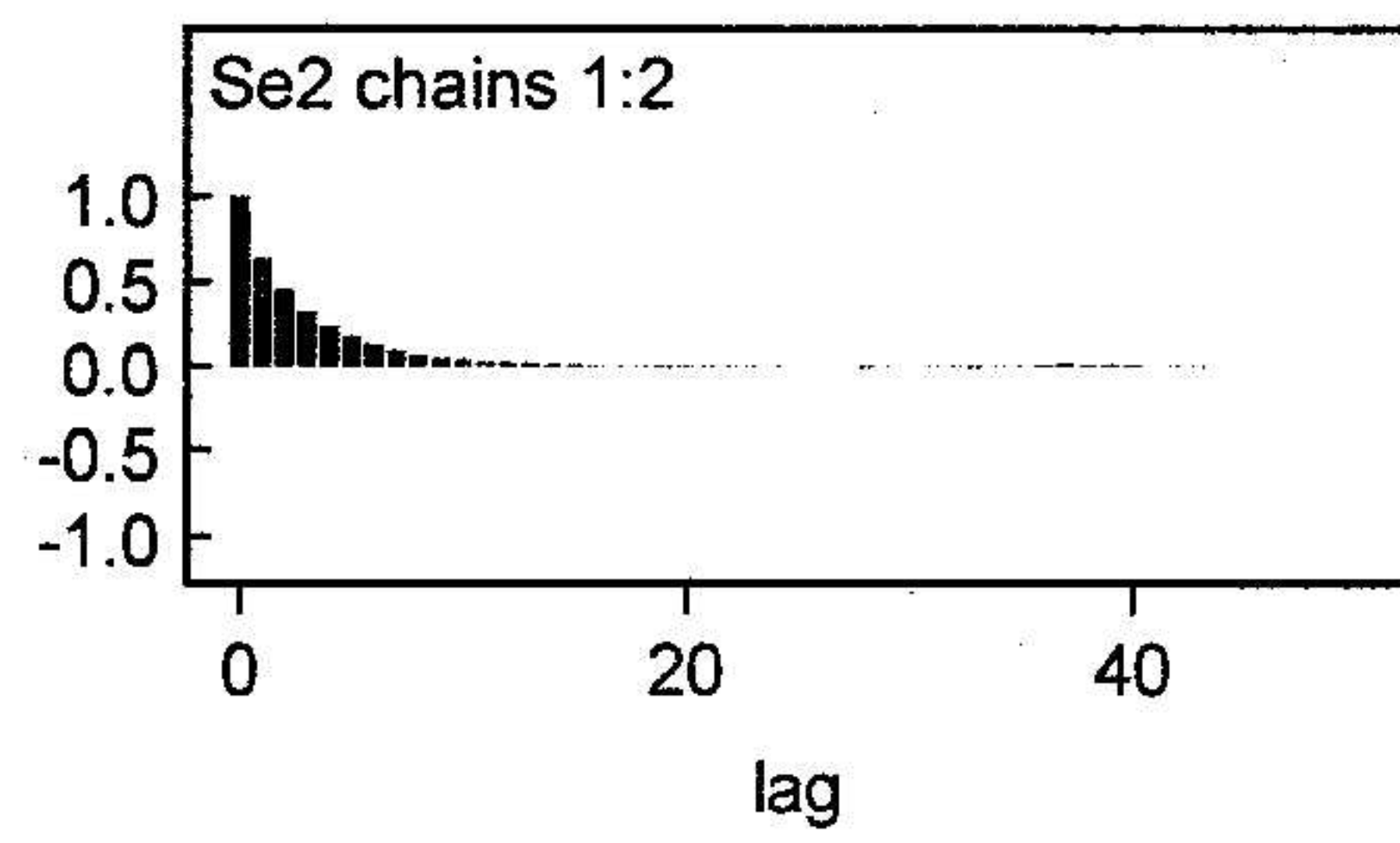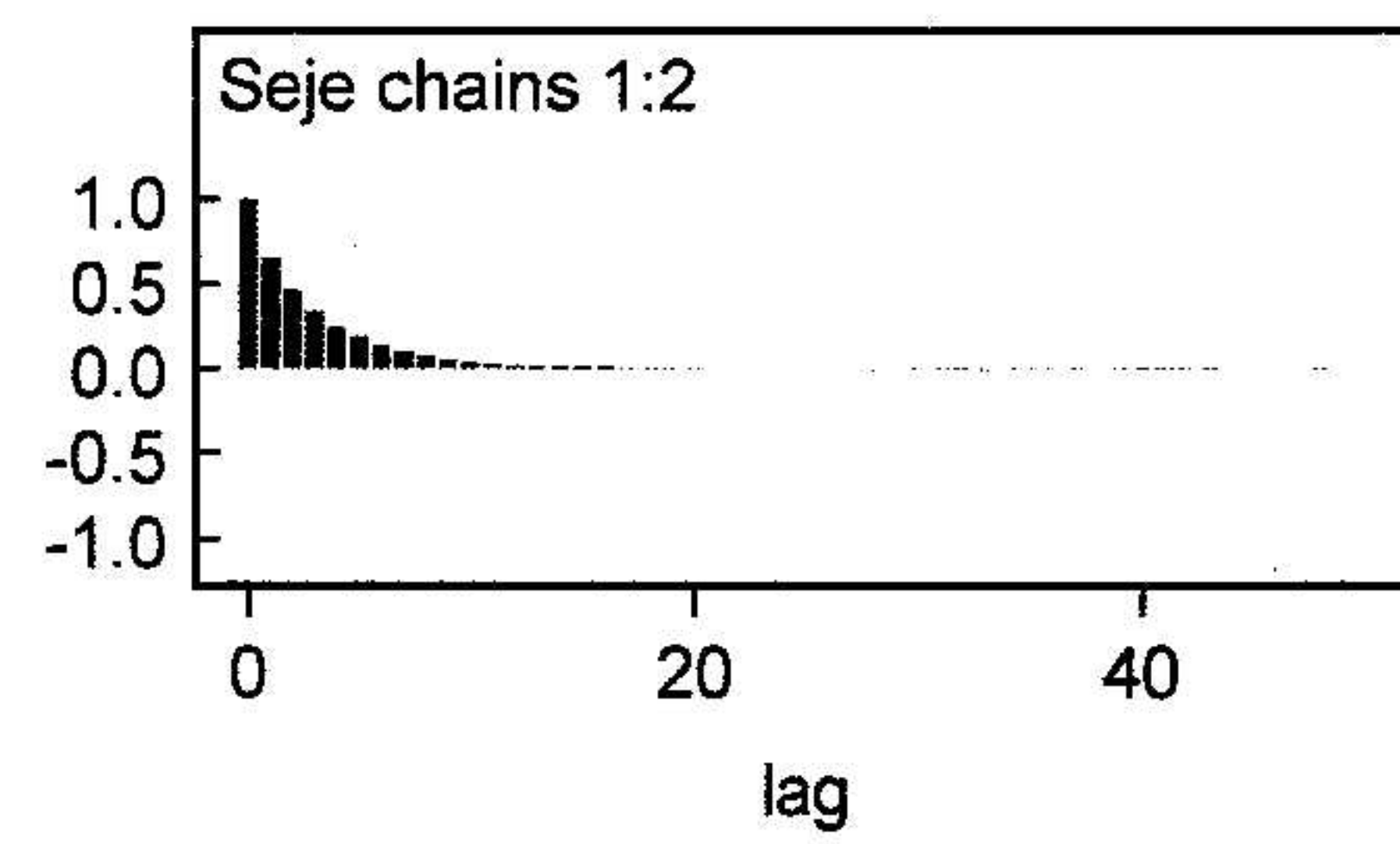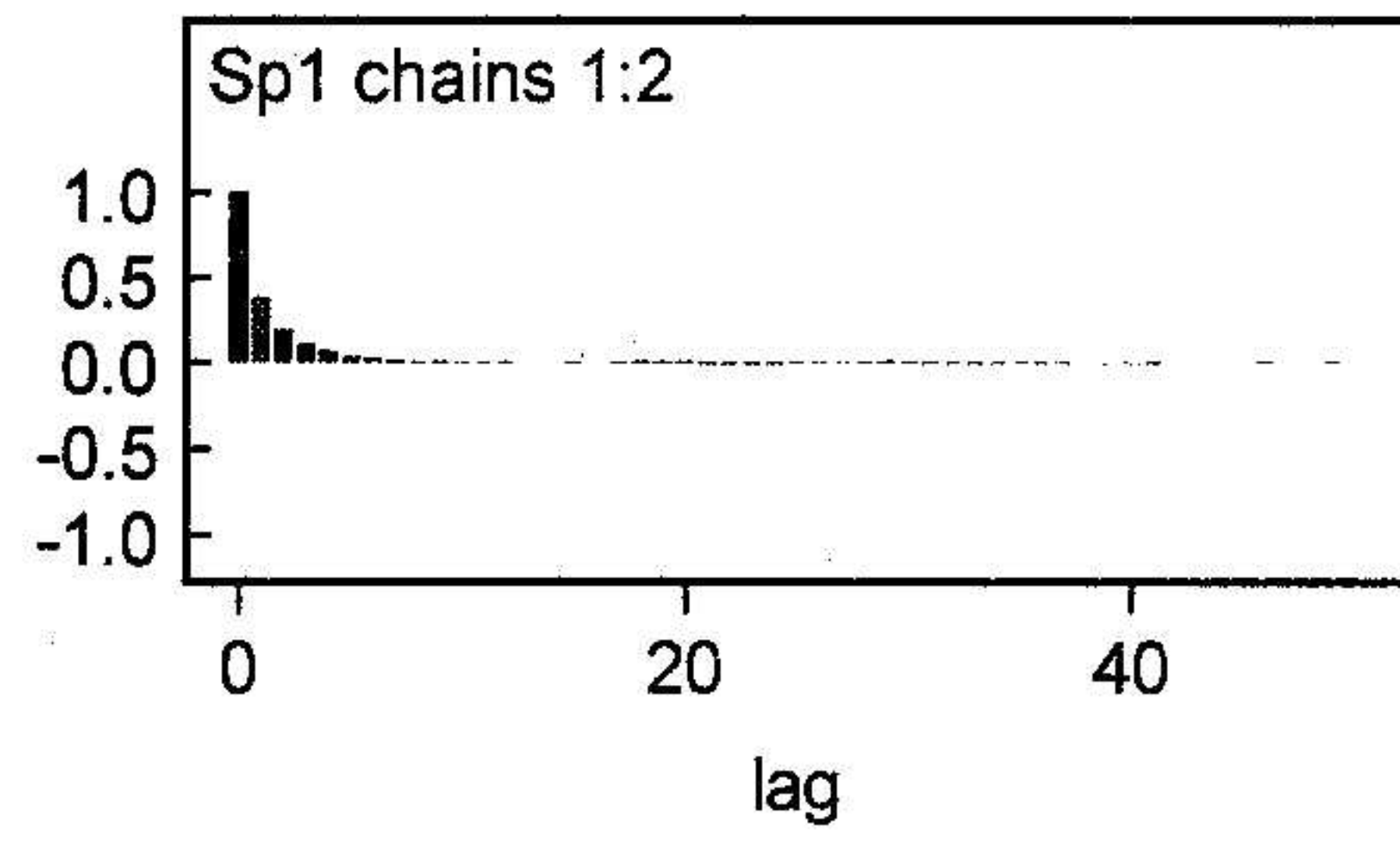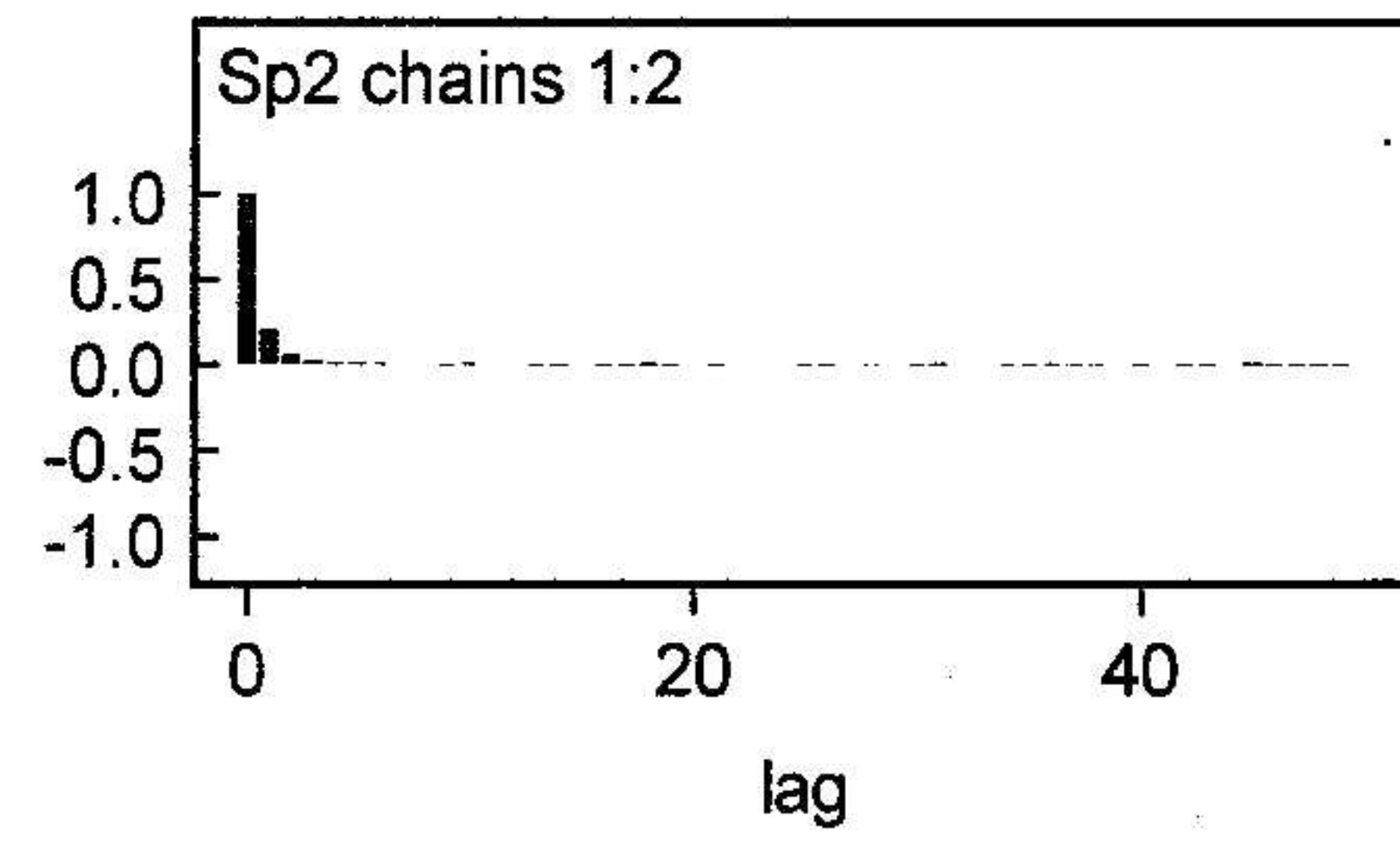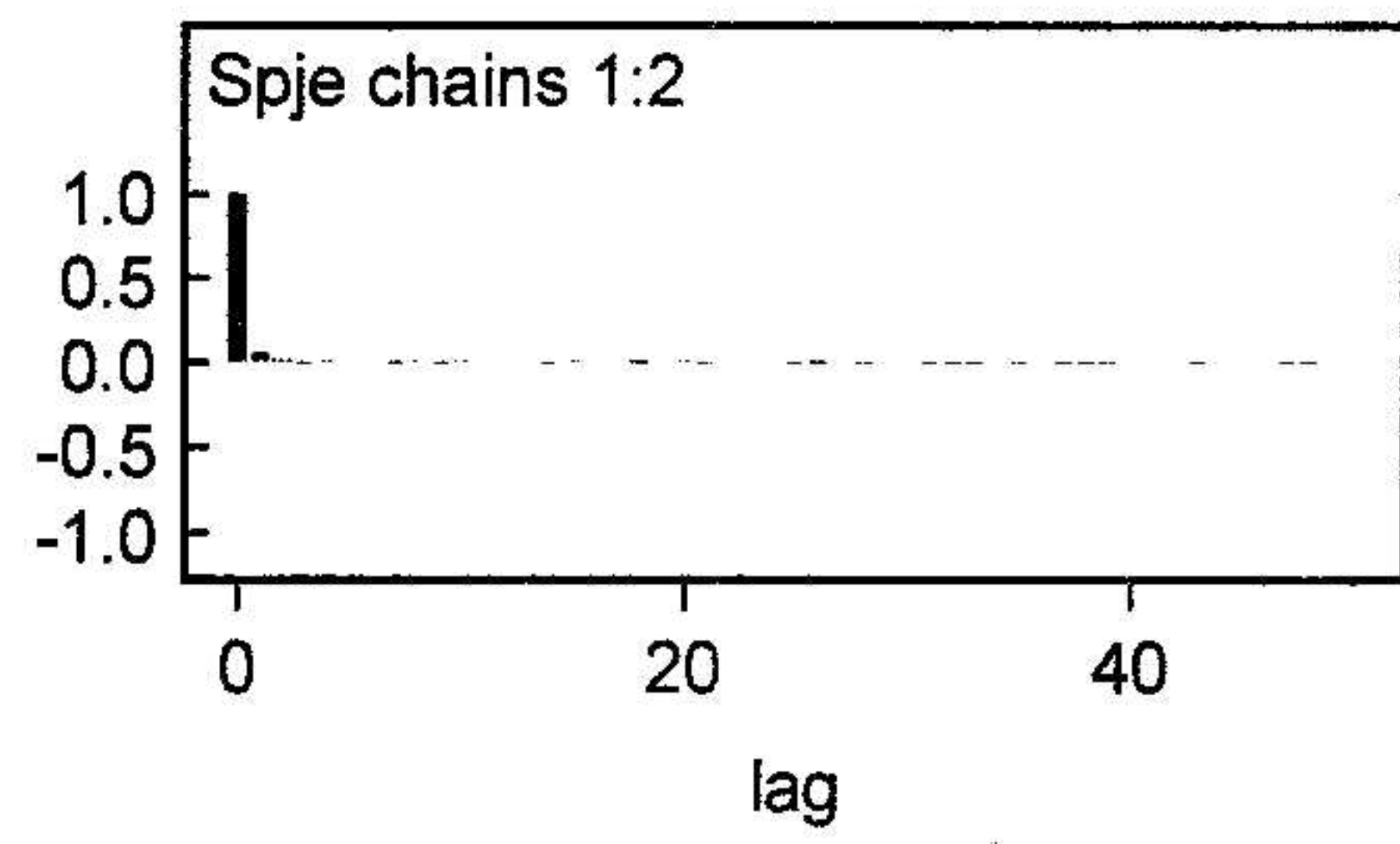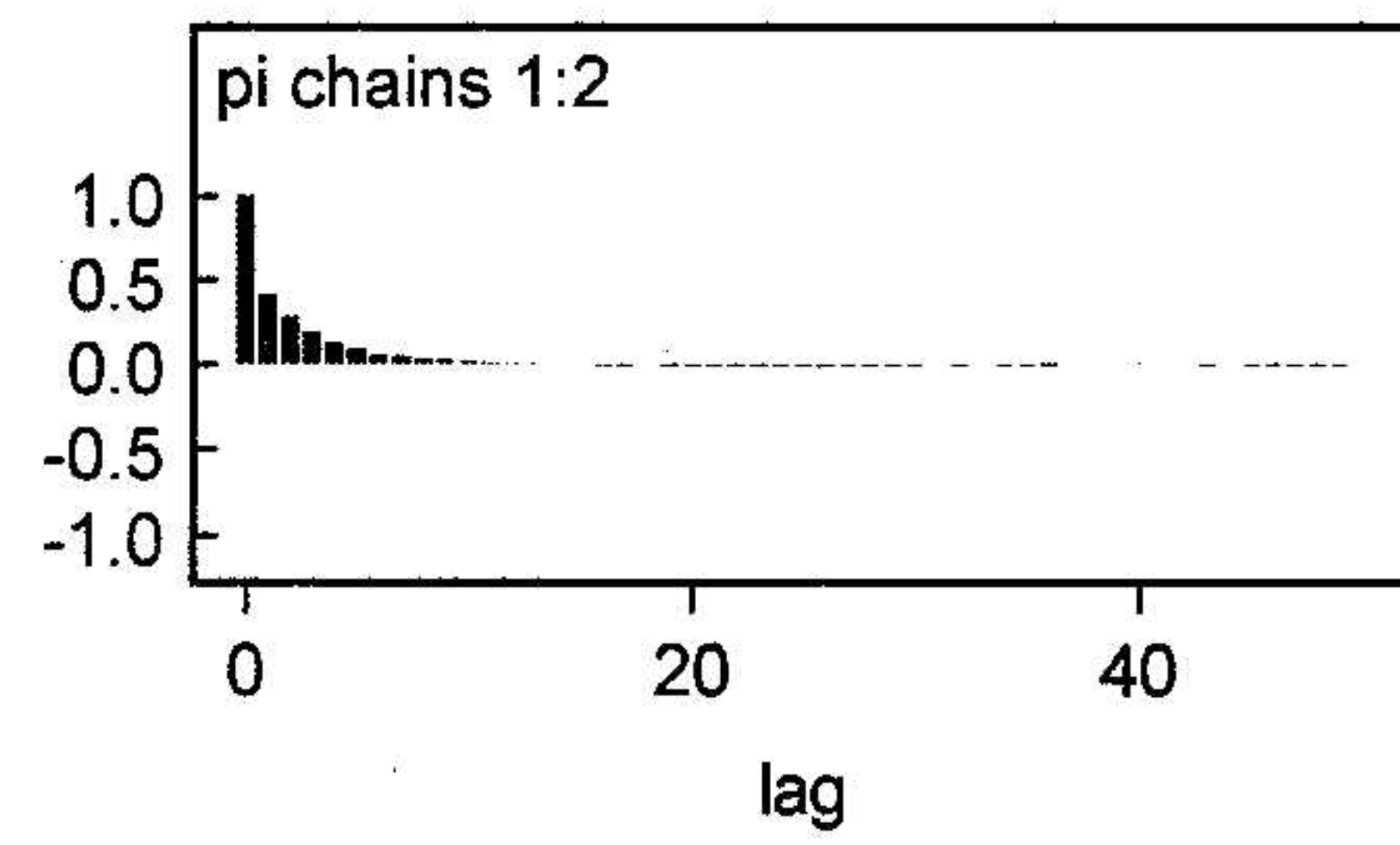

Supplement: Supplementary file 2 — Additional file 2: Figure S1: The convergence diagnosis. (PDF 972 KB) [file 12874_2014_1125_MOESM2_ESM.pdf]
