# Supplementary material for: A two-stage Bayesian method for estimating accuracy and disease prevalence for two dependent dichotomous screening tests when the status of individuals who are negative on both tests is unverified
Source: BMC Med Res Methodol. 2014 Sep 23;14:110. doi: 10.1186/1471-2288-14-110 (PMC4193534; doi:10.1186/1471-2288-14-110)
Supplement: Supplementary file 3 — Additional file 3: The technical details of model evaluation and checking. (DOCX 21 KB) [file 12874_2014_1125_MOESM3_ESM.docx]

**Additional file 3.** The technical details of model evaluation and checking

In this study, we divide the model evaluation and the checking into individual and overall diagnostics. Individual checks are based on the cell in Table 1, and overall diagnostics aim to check the more general assumptions of the model by *DIC* [[21](#_ENREF_21)], *pD* [[5](#_ENREF_5)], and the Chi-squared goodness-of-fit test.

***‘Model key point’ checking***

The ‘model key point’ is the difference between the actual positive predictive value (*PPV*, the proportion of all individuals with a positive test who actually have the disease and the only parameter verified by a gold standard test in these studies) and the model-estimated *PPV*. Based on equation (1), the estimated *PPV* is related to the three basic parameters *Se, Sp*, and *π*. For this reason, it is referred to as the key point in model checking. If the 95% Bayesian credible interval (95% BCI) of the estimated *PPV* covers the actual value, it can be inferred that the model fits the observed value well at the 95% probability level.

***Cell checking***

The differences between the observed and expected frequencies related to *x_1_****_1_****, x_10_, x_01_, x_00_, a_11_,* and *a_10_* in Table 1 are checked. If the observed frequencies are within the 95% BCI, it can be inferred that the model fits the known observation value well at the 95% probability level.

***pD and DIC***

The *pD* is the “effective number of parameters” and measures the approximate effective number of estimated parameters in a fitted statistical model. This measure is no longer an integer because it is calculated as the difference between the posterior mean of the deviance and the deviance evaluated in the posterior mean [[5](#_ENREF_5)]. *DIC* is the “Deviance Information Criterion”. *DIC = Dbar + pD*, where *Dbar* is the posterior mean of the deviance [[30](#_ENREF_30)]. Usually, in Bayesian terms, models with a smaller *DIC* are superior to models with a larger *DIC*. The values of *pD* and *DIC* can be calculated conveniently using WinBUGS [[27](#_ENREF_27)].

***Local Chi-squared goodness-of-fit test***

To evaluate the goodness-of-fit of a model, *χ^2^* and the corresponding *p*-values are the statistics that are used most frequently. The local Pearson *χ^2^* goodness-of-fit test only relates to known observations *x_11_, x_10_, x_01_, x_00_, a_11_*, and *a_10_,* as the negatives in both tests (*a****_0_****_1_*, *a****_0_****_0_*) are unavailable in these types of studies. The calculation of *χ^2^* using WinBUGS can be performed by defining nodes chisq.obs and chisq.rep, which are able to calculate *χ^2^*(*y^obs^, θ^(t)^*) and *χ^2^*(*y^rep,(t)^, θ^(t)^*) in each iteration *t* of the MCMC algorithm, in which *y^obs^* is actual data and *y^rep^* is future (or replicated) data based on predictive distributions. The difference *χ^2^*(*y^rep,(t)^, θ^(t)^*) - *χ^2^*(*y^obs^, θ^(t)^*) and corresponding posterior *p*-value must be monitored, as given by the posterior mean of a node chisq.p as defined in WinBUGS

chisq.p <- step (chisq.rep-chisq.obs)

The binary variable chisq.p takes the value of 1 when *χ^2^*(*y^rep,(t)^, θ^(t)^*) > *χ^2^*(*y^obs^, θ^(t)^*) and zero otherwise. The posterior *p*-values can be directly interpreted as the future probability of observing samples with *D*(*y*, *θ*) higher than the value already observed [[29](#_ENREF_29)]. Values of approximately 0.5 indicate that the distributions of the replicated and actual data are close, whereas values close to zero or one indicate differences between them [[29](#_ENREF_29)].
